# Comparative Genomic Insights into the Evolution of *Halobacteria*-Associated “*Candidatus* Nanohaloarchaeota”

**DOI:** 10.1128/msystems.00669-22

**Published:** 2022-10-19

**Authors:** Dahe Zhao, Shengjie Zhang, Sumit Kumar, Heng Zhou, Qiong Xue, Wurunze Sun, Jian Zhou, Hua Xiang

**Affiliations:** a State Key Laboratory of Microbial Resources, Institute of Microbiology, Chinese Academy of Sciences, Beijing, China; b College of Life Sciences, University of Chinese Academy of Sciences, Beijing, China; c Enzyme and Microbial Biochemistry Lab, Department of Chemistry, Indian Institute of Technology, Hauz Khas, New Delhi, India; d Amity Institute of Biotechnology, Amity University, Noida, Uttar Pradesh, India; University of Pretoria

**Keywords:** “*Candidatus* Nanohaloarchaeota”, DPANN superphylum, symbiosis, horizontal gene transfer, evolution, comparative genomics

## Abstract

Members of the phylum “*Candidatus* Nanohaloarchaeota,” a representative lineage within the DPANN superphylum, are characterized by their nanosized cells and symbiotic lifestyle with *Halobacteria*. However, the development of the symbiosis remains unclear. Here, we propose two novel families, “*Candidatus* Nanoanaerosalinaceae” and “*Candidatus* Nanohalalkaliarchaeaceae” in “*Ca*. Nanohaloarchaeota,” represented by five dereplicated metagenome-assembled genomes obtained from hypersaline sediments or related enrichment cultures of soda-saline lakes. Phylogenetic analyses reveal that the two novel families are placed at the root of the family “*Candidatus* Nanosalinaceae,” including the cultivated taxa. The two novel families prefer hypersaline sediments, and the acid shift of predicted proteomes indicates a “salt-in” strategy for hypersaline adaptation. They contain a lower proportion of putative horizontal gene transfers from *Halobacteria* than “*Ca*. Nanosalinaceae,” suggesting a weaker association with *Halobacteria*. Functional prediction and historical events reconstruction disclose that they exhibit divergent potentials in carbohydrate and organic acid metabolism and environmental responses. Globally, comparative genomic analyses based on the new families enrich the taxonomic and functional diversity of “*Ca*. Nanohaloarchaeota” and provide insights into the evolutionary process of “*Ca*. Nanohaloarchaeota” and their symbiotic relationship with *Halobacteria*.

**IMPORTANCE** The DPANN superphylum is a group of archaea widely distributed in various habitats. They generally have small cells and have a symbiotic lifestyle with other archaea. The archaeal symbiotic interaction is vital to understanding microbial communities. However, the formation and evolution of the symbiosis between the DPANN lineages and other diverse archaea remain unclear. Based on phylogeny, habitat distribution, hypersaline adaptation, host prediction, functional potentials, and historical events of “*Ca*. Nanohaloarchaeota,” a representative phylum within the DPANN superphylum, we report two novel families representing intermediate stages, and we infer the evolutionary process of “*Ca*. Nanohaloarchaeota” and their *Halobacteria*-associated symbiosis. Altogether, this research helps in understanding the evolution of symbiosis in “*Ca*. Nanohaloarchaeota” and provides a model for the evolution of other DPANN lineages.

## INTRODUCTION

The DPANN superphylum (an acronym of five candidate phylum names, “*Candidatus* Diapherotrites,” “*Candidatus* Parvarchaeota,” “*Candidatus* Aenigmarchaeota,” “*Candidatus* Nanoarchaeota,” and “*Candidatus* Nanohaloarchaeota”) is a group of archaea with nanosized cells and small genomes (1–3). Despite different classifications in the Genome Taxonomy Database (GTDB) and NCBI taxonomy database (4), more and more lineages are classified in the DPANN superphylum, including “*Candidatus* Micrarchaeota” (5), “*Candidatus* Woesearchaeota” (6), “*Candidatus* Pacearchaeota” (6), “*Candidatus* Huberarchaeota” (7), “*Candidatus* Mamarchaeota” (8), and “*Candidatus* Undinarchaeota” (9). The DPANN group formed at the early stage of archaeal evolution (9–13). A general symbiotic lifestyle is proposed from the reduced metabolic potentials of most members (3, 5, 6, 14). The symbiosis was demonstrated based on the cocultures of DPANN lineages and their hosts (2, 15–19). Many lineages, like “*Ca*. Nanoarchaeota,” “*Ca*. Huberarchaeota,” “*Ca*. Nanohaloarchaeota,” and “*Ca*. Aenigmarchaeota,” were predicted to exchange genes with their respective hosts via horizontal gene transfer (HGT) (9, 19–22). However, it remains unknown how the DPANN lineages form symbioses with diverse taxa.

“*Ca*. Nanohaloarchaeota” is one of the first five phyla in the DPANN group (1). This phylum is widely distributed in (hyper)saline habitats (3, 23–27) by harnessing the energetically favorable “salt-in” strategy (23, 25) like their host *Halobacteria* (28). “*Ca*. Nanohaloarchaeota” cells were revealed to have a tight symbiotic relationship with the class *Halobacteria*, as demonstrated by their cocultures (17, 18). In some “*Ca*. Nanohaloarchaeota” genomes, long “SPEARE” proteins containing serine protease, adhesion, and restriction endonuclease domains were supposed to function in attachment and invasion of hosts (17). Remarkably, the nanohaloarchaeon “*Candidatus* Nanohalobium constans” LC1Nh exhibits mutualistic symbiosis with the host under conditions with glycogen or starch as a carbon source (18). “*Ca*. Nanohaloarchaeota” shares 21% of sisterhood relationships with *Halobacteria* (9), and similar HGT events have also been reported (22). However, because of the protein adaptation to high salinity in the cytoplasm, some of the close relationships may be the result of compositional biases from convergent evolution (9, 23, 25). Based on the cocultivation and genomic prediction, “*Ca*. Nanohaloarchaeota” were considered aerotolerant anaerobes with a lifestyle of sugar fermentation, while the hosts generally perform aerobic respiration (3, 18, 23, 24). In addition, all these reports on “*Ca*. Nanohaloarchaeota” were focused on one family, i.e., “*Candidatus* Nanosalinaceae” (see Results and Discussion).

In this research, we report five dereplicated metagenome-assembled genomes (MAGs) of two novel families named “*Candidatus* Nanoanaerosalinaceae” and “*Candidatus* Nanohalalkaliarchaeaceae” in “*Ca*. Nanohaloarchaeota.” They were obtained from hypersaline sediments or enrichment cultures from soda-saline lakes. Furthermore, we performed comparative genomic analyses on habitat distribution, amino acid composition, and functional gene prediction. These results provide insights into the evolution of “*Ca*. Nanohaloarchaeota” symbiosis with *Halobacteria*.

## RESULTS AND DISCUSSION

### Acquisition of the genomes of two novel families within the phylum “*Ca*. Nanohaloarchaeota.”

We performed metagenomic analyses on enrichment cultures of five deep sediments of a soda-saline lake in Inner Mongolia, China (described in Materials and Methods), and reanalyzed our previously reported metagenomes of brine and sediment samples from the same natural environments (24, 29). In this research, we obtained 10 MAGs affiliated with “*Ca*. Nanohaloarchaeota” (see Table S1 in the supplemental material). In the description below, we mainly follow the GTDB taxonomy unless otherwise specified. The taxonomic annotation based on the GTDB database (release 202) reveals that all 10 genomes as well as 16 “*Ca*. Nanohaloarchaeota” genomes deposited in public databases belong to the order “*Candidatus* Nanosalinales” (Table S1), of which 19 (five are obtained in this study) are affiliated with the family “*Ca*. Nanosalinaceae,” and the other 7 are not classified (including the two novel families described below).

10.1128/msystems.00669-22.6 TABLE S1 Statistical description of the genomes of “*Ca*. Nanohaloarchaeota,” “*Ca*. Aenigmatarchaeota,” and three unidentified phyla. Download Table S1, XLSX file, 0.04 MB. Copyright © 2022 Zhao et al. 2022 Zhao et al. https://creativecommons.org/licenses/by/4.0/ This content is distributed under the terms of the Creative Commons Attribution 4.0 International license.

**FIG 1:**
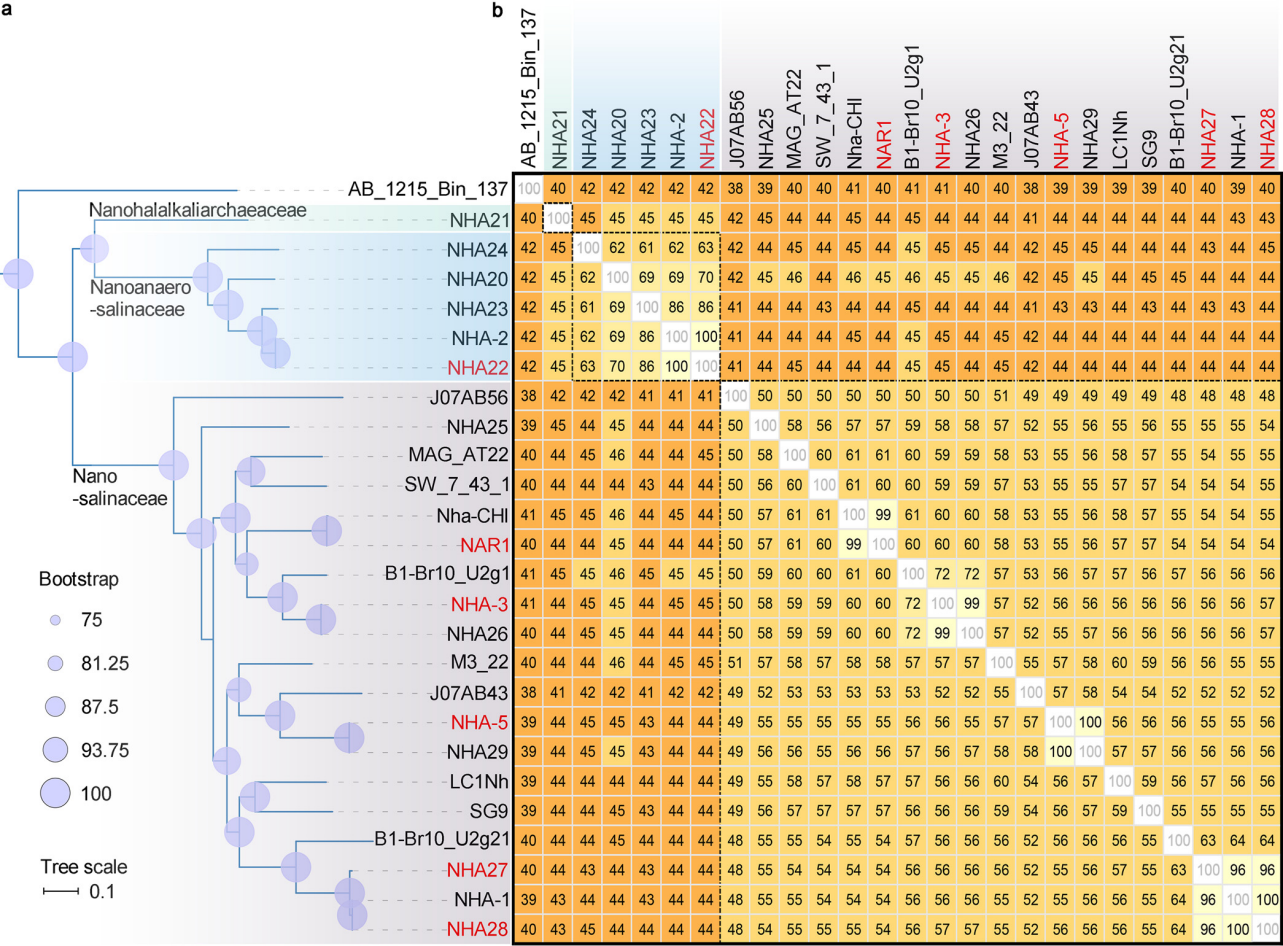
Phylogeny of the phylum “*Ca*. Nanohaloarchaeota.” (a) Phylogenomic tree based on the 122 single-copy ubiquitous proteins in GTDB. It was obtained by pruning the tree in Fig. S1. Briefly, the best-fit model of LG+F+G4 was chosen, and a consensus tree based on ultrafast bootstrap approximation of 1,000 times is presented. (b) Average AAI matrix for the genomes of “*Ca*. Nanohaloarchaeota.” The data are rounded by omitting decimal fractions smaller than 0.5 and counting all others (including 0.5) as 1. The background (from yellow to orange) is colored according to the threshold AAIs of species, genus, and family (95.0, 65.0, and 45.0%, respectively). The genomes sharing an AAI of more than 95.0% with other genomes of higher completeness or low contamination are marked by red, and they were abandoned in subsequent research.

In the phylogenetic tree based on the 122 single-copy conserved proteins in the GTDB, the phylum “*Ca*. Nanohaloarchaeota” members are closely related to EX4484-52, “*Ca*. Aenigmatarchaeota,” PWEA01, and QMZS01 (see Fig. S1 in the supplemental material), and the five phyla (“*Ca*. Nanohaloarchaeota,” “*Ca*. Aenigmatarchaeota,” and three unidentified phyla [NATU]) are located in the DPANN group (see Fig. S6 at https://doi.org/10.6084/m9.figshare.19549495). The phylum “*Ca*. Nanohaloarchaeota” is classified into four family-level lineages with bootstrap supports of more than 75%, including “*Ca*. Nanosalinaceae,” “*Ca*. Nanoanaerosalinaceae,” “*Ca*. Nanohalalkaliarchaeaceae,” and AB_1215_Bin_137 (Fig. 1a). The five representative MAGs of novel families are shown in Table 1. In fact, AB_1215_Bin_137 is a MAG obtained from Guaymas Basin (Gulf of California) sediment samples (30). It is classified as a member of the order “*Ca*. Nanosalinales” by GTDB-Tk (Table S1), but it has a relatively long distance from the other three families (Fig. 1a), and its 16S rRNA gene shares similarities of less than 82.0% (order threshold) (31) with them (Fig. S2a). Therefore, it may represent a novel order. The families “*Ca*. Nanoanaerosalinaceae” and “*Ca*. Nanohalalkaliarchaeaceae” are placed at the root of “*Ca*. Nanosalinaceae” (Fig. 1a). The average amino acid identity (AAI) values of the predicted proteome between the genomes of the families “*Ca*. Nanoanaerosalinaceae” and “*Ca*. Nanosalinaceae” are generally less than 45.0%, the family boundary according to the previous literature (32); those among genomes of the same family are generally greater than 45.0% (Fig. 1b). The 16S rRNA gene identity analysis backs the classification of “*Ca*. Nanoanaerosalinaceae” (Fig. S2a) according to the taxonomic thresholds of sequence identities for order and family (82.0% and 86.5%, respectively) (31). Similarly, the MAG NHA21 of the family “*Ca*. Nanohalalkaliarchaeaceae” has AAI with “*Ca*. Nanosalinaceae” below 45% but approximately 45% AAI with “*Ca*. Nanoanaerosalinaceae” (Fig. 1b). Although no 16S rRNA gene is annotated in NHA21 for further evidence, considering the significantly different donor profile of HGT and average isoelectric point (ApI) from “*Ca*. Nanoanaerosalinaceae” (described in “Symbiotic host prediction of the two novel families” and “Acidic proteomes of the two novel families for hypersaline adaptation”), we recommend the novel family “*Ca*. Nanohalalkaliarchaeaceae” represented by NHA21. Phylogenetic analyses based on ribosome proteins and the 16S rRNA gene are in good line with the taxonomy of “*Ca*. Nanohaloarchaeota” (Fig. S7 to S10 [https://doi.org/10.6084/m9.figshare.19549495]). In conclusion, we propose two new families which form a branch in the phylogenetic trees and are located at the root of the “*Ca*. Nanosalinaceae.” Of all 26 “*Ca*. Nanohaloarchaeota” genomes (10 obtained in this study), six shared more than 95% AAI and average nucleotide identity (ANI) for species boundaries (32), and they were removed in further research for lower completeness or higher contamination (Fig. 1; Fig. S2b). We observed that all 26 “*Ca*. Nanohaloarchaeota” genomes had low completeness values of 70.40 to 87.31% (Table S1), but a representative member with a higher completeness for each species could be reasonably selected.

**TABLE 1:** Genomic features of representative MAGs affiliated with “*Ca*. Nanoanaerosalinaceae” and “*Ca*. Nanohalalkaliarchaeaceae”

MAG	WGS accession no.	Size (bp)	No. of contigs	*N*_50_ (bp)	GC (mol%)	Completeness (%)	Contamination (%)	Taxonomy at family level	Reference
NHA21	JALDAF000000000	1,130,266	78	19,632	56.09	78.94	0.00	*Nanohalalkaliarchaeaceae*	This study
NHA20	JALDAE000000000	840,242	60	21,270	41.20	79.91	0.00	*Nanoanaerosalinaceae*	This study
NHA23	JALDAH000000000	849,024	82	17,982	38.05	78.50	0.93	*Nanoanaerosalinaceae*	This study
NHA24	JALDAI000000000	762,957	106	9,734	38.12	76.53	2.80	*Nanoanaerosalinaceae*	This study
NHA-2	JAKOOZ000000000	876,291	78	14,011	38.52	81.00	0.93	*Nanoanaerosalinaceae*	24

10.1128/msystems.00669-22.1 FIG S1 Phylogenomic analysis of NATU-lineage genomes based on the concatenated 122 single-copy ubiquitous proteins in GTDB. The model LG+F+G4 was chosen according to the Bayesian information criterion. Bootstrap (percent) supports based on an ultrafast bootstrap approximation of 1,000 are shown at the nodes. NATU, “*Ca*. Nanohaloarchaeota”, “*Ca*. Aenigmatarchaeota”, and three unidentified phyla. Branch colors are as follows: red, EX4484-52; blue, “*Ca*. Nanohaloarchaeota”; green, PWEA01; violet, QMZS01; orange, “*Ca*. Aenigmatarchaeota.” Download FIG S1, TIF file, 0.9 MB. Copyright © 2022 Zhao et al. 2022 Zhao et al. https://creativecommons.org/licenses/by/4.0/ This content is distributed under the terms of the Creative Commons Attribution 4.0 International license.

10.1128/msystems.00669-22.2 FIG S2 Identity analyses of 16S rRNA genes and genomes in “*Ca*. Nanohaloarchaeota.” (a) Identity percentage of 16S rRNA genes from “*Ca*. Nanohaloarchaeota.” The locus tags of sequences are listed in Table S1. The background (from yellow to red) is colored according to the threshold sequence identities of species (98.7%), genus (94.5%), family (86.5%), order (82.0%), and class (78.5%). Some strains (including NHA21) are not shown, because no 16S rRNA gene was found in their genomes. The data are rounded by omitting decimal fractions smaller than 0.5 and counting all others (including 0.5) as 1. (b) Percent ANI. NA, ANI value is less than 70%. ANI values above 95% are in red and marked by a pink background. Download FIG S2, TIF file, 2.1 MB. Copyright © 2022 Zhao et al. 2022 Zhao et al. https://creativecommons.org/licenses/by/4.0/ This content is distributed under the terms of the Creative Commons Attribution 4.0 International license.

Markedly, the taxonomy is different from the previous report (18), in which the taxa of the family “*Ca*. Nanosalinaceae” were classified into three classes. The possible reason may be that the other lineages placed at the root of “*Ca*. Nanohaloarchaeota” were not included in that phylogenomic analysis. In addition, we found that some “*Ca*. Nanohaloarchaeota” genomes are incorrectly classified in the NCBI taxonomy database. Ten assemblies were in the “*Ca*. Nanohaloarchaeota” (under taxid 1462430) of the DPANN group, but 29 were in the class “*Ca*. Nanohaloarchaea” (under taxid 1051663) of *Euryarchaeota*. It is clear that they share a very close relationship with each other. The misinterpretation was considered the result of inadequate outgroup representation (1). Moreover, the well-researched “*Ca*. Nanohaloarchaeota” taxa belong to the family “*Ca*. Nanosalinaceae” (Fig. 1), including the two cocultures with *Halobacteria* (17, 18). In addition, the class *Halobacteria* (as host of “*Ca*. Nanohaloarchaeota”) is affiliated with the phylum *Halobacteriota* (Fig. S6 to S8 [https://doi.org/10.6084/m9.figshare.19549495]), whose subordinates belong to the phylum *Euryarchaeota* of the classical taxonomy (4).

### The two novel families prefer the habitats of hypersaline sediment.

The five representative genomes affiliated with the novel families “*Ca*. Nanoanaerosalinaceae” and “*Ca*. Nanohalalkaliarchaeaceae” were obtained from hypersaline and alkaline sediment samples or the related enrichment cultures (Table S6 [https://doi.org/10.6084/m9.figshare.19549495]). They seem to have a habitat preference different from that of “*Ca*. Nanosalinaceae,” which were generally reported to occur in hypersaline brines (3, 23, 24). Therefore, the relative abundance of the 20 dereplicated “*Ca*. Nanohaloarchaeota” genomes was estimated in the metagenomes of soda-saline lake samples (24, 29) or the enrichment cultures (this study) with different salinities.

**FIG 2:**
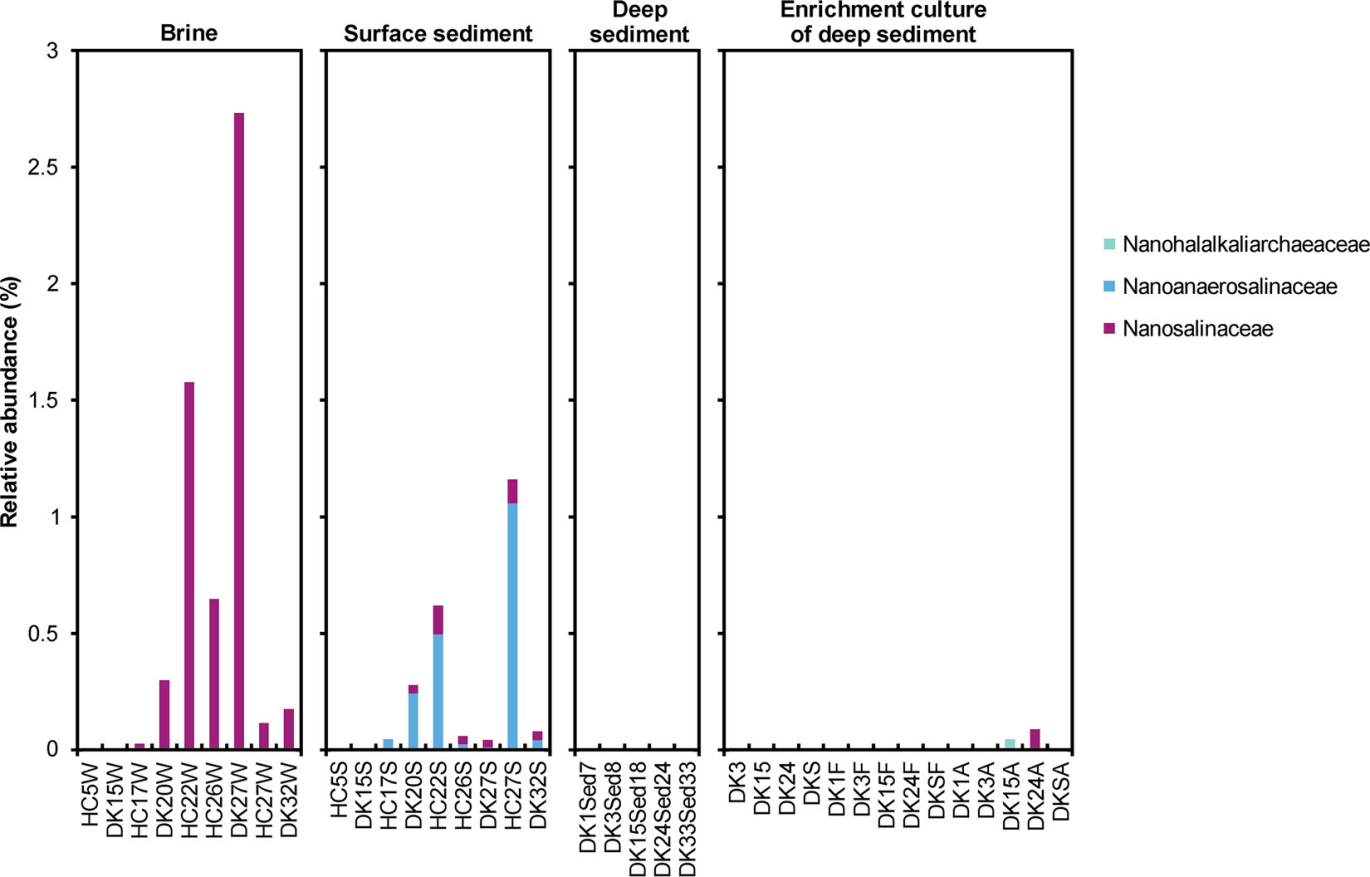
Relative abundance of the three families of the phylum “*Ca*. Nanohaloarchaeota” in soda-saline lake samples and the related enrichment cultures. The relative abundance was estimated based on the percentage of the reads that mapped onto the MAG obtained from the soda-saline lake samples, because most MAGs assembled from other samples were not detected (Table S2). NHA21, “*Ca*. Nanohalalkaliarchaeaceae”; NHA24, NHA20, NHA23, and NHA-2, “*Ca*. Nanoanaerosalinaceae”; NHA25, NHA26, NHA29, and NHA-1, “*Ca*. Nanosalinaceae.” The metagenomes of brine, surface sediment, and deep sediment were published in previous reports (24, 29). In their sample names, HC and DK represent Hutong Qagan Lake and Habor Lake, respectively, and the numbers represent the salinities of brines; in the brine and surface sediment, W and S represent water (or brine) and sediment, respectively; in the deep sediment, the numbers following “Sed” show the salinities of the pore water. The enrichment culture is described in Materials and Methods.

On the whole, almost all the MAGs not obtained from our samples could not be detected (Table S2). Therefore, we counted only the MAGs that were assembled from our soda-saline lake samples. The result shows that “*Ca*. Nanosalinaceae” are detected predominantly in the hypersaline brine (more than 20% salinity) and marginally in the hypersaline surface sediment (Fig. 2). The detection of “*Ca*. Nanosalinaceae” in the surface sediment might be the result of brine interfusion. Therefore, this result is mainly in agreement with the previous reports (3, 23, 24). MAG NHA25 was an exception detected in the enrichment culture of hypersaline sediment with acetate added, and a probable cause might be that it did not harbor the *SOD2* gene (discussed below).

10.1128/msystems.00669-22.7 TABLE S2 Relative abundance of 20 dereplicated MAGs of “*Ca*. Nanohaloarchaeota” in soda-saline lake metagenomes. Download Table S2, XLSX file, 0.01 MB. Copyright © 2022 Zhao et al. 2022 Zhao et al. https://creativecommons.org/licenses/by/4.0/ This content is distributed under the terms of the Creative Commons Attribution 4.0 International license.

Apparently, the “*Ca*. Nanoanaerosalinaceae” are found in hypersaline surface sediment but not in brine and deep sediment samples, while “*Ca*. Nanohalalkaliarchaeaceae” are detected in the enrichment cultures of deep sediment (Fig. 2; Table S2). In fact, the metagenome exhibits a snapshot of the true microbial community. The mixture of deep sediment samples of four sites from each pond could reduce the spatial heterogeneity (29), but some “*Ca*. Nanohaloarchaeota” taxa were reported to experience a diel cycle in relative abundance (33). Consequently, considering the fact that the enrichment cultures are under anaerobic condition and from deep sediment, “*Ca*. Nanohalalkaliarchaeaceae” could be inferred to inhabit hypersaline deep sediment, although they were not detected in the natural environments (Fig. 2; Table S2). Overall, the three families seemingly prefer different hypersaline habitats; i.e., “*Ca*. Nanosalinaceae,” “*Ca*. Nanoanaerosalinaceae,” and “*Ca*. Nanohalalkaliarchaeaceae” colonized brine, surface sediment, and deep sediment, respectively. Notably, our samples are from saline and alkaline environments. However, “*Ca*. Nanoanaerosalinaceae” and “*Ca*. Nanohalalkaliarchaeaceae” might also exist in neutral hypersaline sediment, in view of the presence of the family “*Ca*. Nanosalinaceae” in both alkaline and neutral brine (23–25, 27). The investigation of neutral hypersaline sediment is necessary to answer this question.

### Symbiotic host prediction of the two novel families.

**FIG 3:**
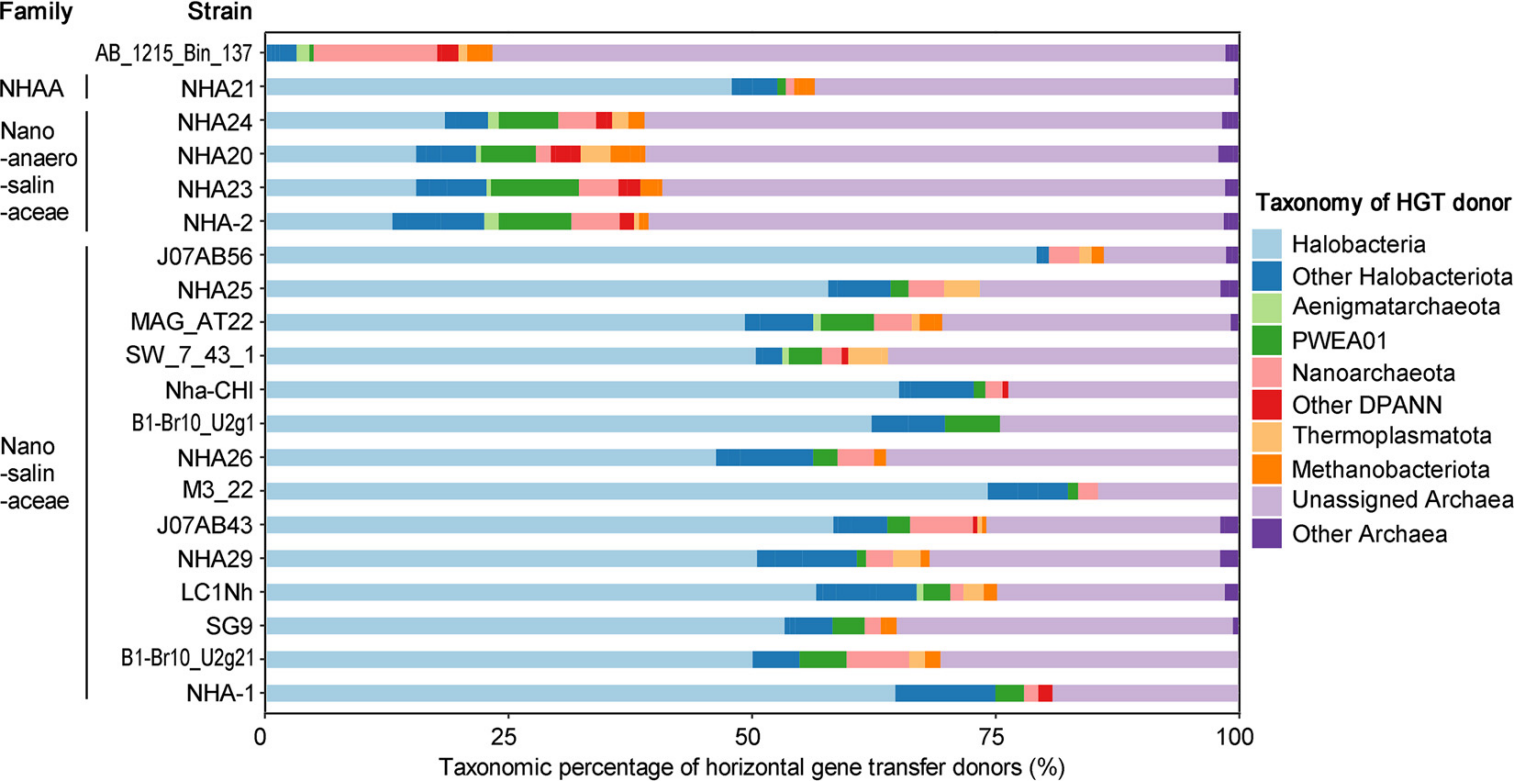
Horizontal gene transfer inference in the genomes of the phylum “*Ca*. Nanohaloarchaeota.” The taxonomic percentage of horizontal gene transfer donors for each strain was inferred using the HGTector pipeline with a GTDB taxonomy-based database based on the archaeal genomes in GTDB release 202. The genomes that are affiliated with the families “*Ca*. Nanosalinaceae,” “*Ca*. Nanoanaerosalinaceae,” and “*Ca*. Nanohalalkaliarchaeaceae” (NHAA) are marked.

Considering the gene exchange between “*Ca*. Nanosalinaceae” and *Halobacteria* as a result of their close association with each other in natural habitats (9), we predicted putative HGTs using the HGTector tool, which is founded on sequence homology search hit distribution statistics (34). First, we created a GTDB archaeal taxonomy-based database (described in Materials and Methods), because the default database follows NCBI taxonomy, in which 29 genomes were incorrectly classified in “*Ca*. Nanohaloarchaea” (taxid 1051663) of *Euryarchaeota*. Afterward, the HGT events were predicted in the 14 representative genomes of the family “*Ca*. Nanosalinaceae” by following the tutorials. The result shows that HGT events could be found in them (Table S9 [https://doi.org/10.6084/m9.figshare.19549495]), and these genomes harbor high proportions (46.25 to 79.25%) of genes horizontally acquired from *Halobacteria* (Fig. 3). Although the tools are different, a conclusion similar to that in the previous report could be drawn (9).

Furthermore, this database was used for HGT prediction of other lineages. The result reveals that NHA21 of “*Ca*. Nanohalalkaliarchaeaceae” harbors 47.86% of horizontally acquired genes from *Halobacteria*. Although “*Ca*. Nanosalinaceae” acquire significantly higher percentages of horizontally transferred genes from *Halobacteria* than NHA21, as estimated using the *t* test (*P* < 0.05) (details shown in Text S1 in the supplemental material), we still believe that NHA21 may keep an associated connection with *Halobacteria*, because the proportion of HGTs, i.e., almost half, was considered high. Another clue is that most of the other half of horizontally acquired genes are from unassigned *Archaea* (Fig. 3), indicating that the symbiosis plays a role almost equal to that of the natural environment as the source of gene gains in NHA21. The class *Halobacteria* were generally reported to be predominant in the brine of saline lake or saltern (23, 25), while it also exhibited high abundance in some hypersaline sediments (35, 36), including soda-saline sediment in our previous research (24, 29). Some *Halobacteria* have the capability of strict or facultative anaerobic respiration based on elemental sulfur, dimethyl sulfoxide (DMSO), or nitrate (37–40). The diverse anaerobic respirations provide a metabolic basis for the presence of *Halobacteria* in the hypersaline sediment. Although it is difficult to predict the detailed taxon of *Halobacteria* for NHA21 as hosts, our data revealed at least one indication that NHA21 might form close interactions with *Halobacteria*.

10.1128/msystems.00669-22.10 TEXT S1 Outputs for statistical analysis of mean comparison. Download Text S1, PDF file, 0.3 MB. Copyright © 2022 Zhao et al. 2022 Zhao et al. https://creativecommons.org/licenses/by/4.0/ This content is distributed under the terms of the Creative Commons Attribution 4.0 International license.

In “*Ca*. Nanoanaerosalinaceae,” only 12.94 to 18.33% of the HGT events are found from *Halobacteria* (Fig. 3), significantly lower than “*Ca*. Nanosalinaceae” and “*Ca*. Nanohalalkaliarchaeaceae” (Text S1). Compared to none of the 228 HGT events in AB_1215_Bin_137 detected with *Halobacteria* as donors, “*Ca*. Nanoanaerosalinaceae” members harbor moderate proportions of horizontally acquired genes from *Halobacteria* (Fig. 3; Table S9 [https://doi.org/10.6084/m9.figshare.19549495]). However, the main donors of horizontally acquired genes in “*Ca*. Nanoanaerosalinaceae” genomes were predicted as unassigned archaea over *Halobacteria* (Fig. 3). This suggests that the environment predominantly shapes the genomic features. Even so, considering the general symbiosis in DPANN (2, 3, 5, 6, 14–19), limited metabolic potentials in “*Ca*. Nanoanaerosalinaceae” (see Fig. 5; Table S4, described below), and a slightly higher proportion of HGTs from *Halobacteria* than other *Halobacteriota*, PWEA01, and “*Ca*. Nanoarchaeota” (Fig. 3), we infer that *Halobacteria* is the most probable host for “*Ca*. Nanoanaerosalinaceae,” but the connection might be not so close as that of “*Ca*. Nanosalinaceae” and “*Ca*. Nanohalalkaliarchaeaceae.”

10.1128/msystems.00669-22.9 TABLE S4 Functional annotation of the representative genomes in the phylum “*Ca*. Nanohaloarchaeota”. Download Table S4, XLSX file, 0.04 MB. Copyright © 2022 Zhao et al. 2022 Zhao et al. https://creativecommons.org/licenses/by/4.0/ This content is distributed under the terms of the Creative Commons Attribution 4.0 International license.

### Acidic proteomes of the two novel families for hypersaline adaptation.

**FIG 4:**
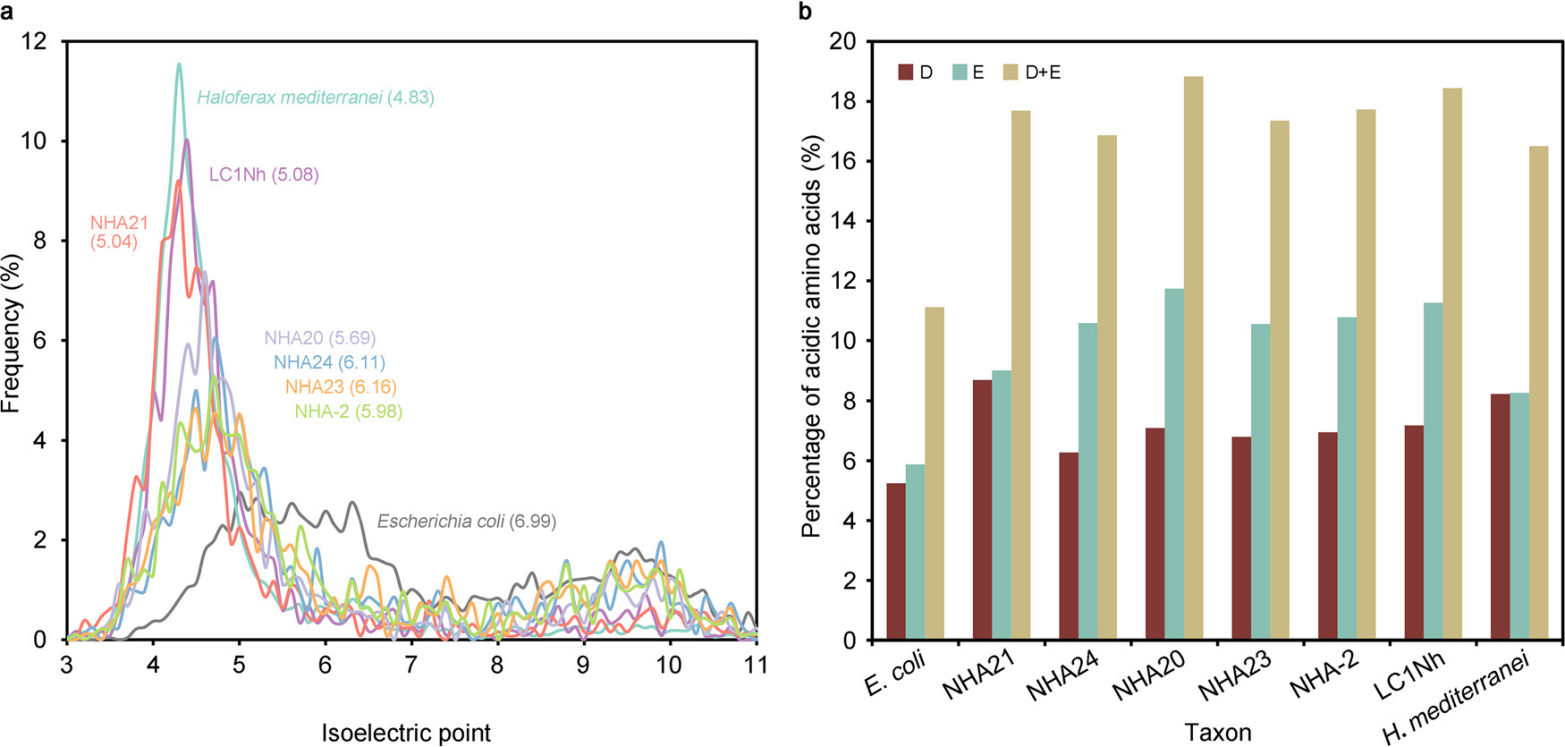
Comparison of isoelectric point profiles and amino acid compositions. (a) Isoelectric point profiles of the predicted proteomes of “*Ca*. Nanoanaerosalinaceae,” “*Ca*. Nanohalalkaliarchaeaceae,” and reference species. The *y* axis shows the frequencies of proteins in the proteomes at each isoelectric point. The isoelectric point of each protein was predicted based on the amino acid sequence. The isoelectric point profiles with a bin width of 0.1 are shown. Haloferax mediterranei ATCC 33500 (GCA_000306765.2) and Escherichia coli O157:H7 strain Sakai (GCA_000008865.2) represent acid-shifted salt-in halophiles and nonhalophiles, respectively. Numbers in parentheses are average isoelectric points (more details are presented in Table S3). (b) Percentage of acidic amino acid glutamate, aspartate, and both. The composition was calculated from the predicted proteome based on the genome sequence. D, aspartate; E, glutamate; D+E, the sum of glutamate and aspartate.

It is widely believed that “*Ca*. Nanosalinaceae” members adopt the “salt-in” strategy to resist the high osmotic pressure of hypersaline environments (23, 25), while AB_1215_Bin_137 inhabiting deep-sea sediment (30) may not be obligated to adapt to the extreme osmotic pressure. During the evolution of the salt-in strategy, the isoelectric point profiles of the predicted proteome became acid shifted (23, 25), because the negatively charged amino acids can maintain the stability and activity of proteins under hypersaline conditions (41). Consequently, we compared the isoelectric point profiles and amino acid compositions of the predicted proteomes of the two novel families and reference lineages. Our results support the idea that the isoelectric point profiles of the predicted proteomes of the family “*Ca*. Nanosalinaceae” were acid shifted (Fig. S3), and their average isoelectric points range from 4.87 to 5.59 (Table S3). They are close to the three *Halobacteria* references (4.71 to 4.83). Notably, AB_1215_Bin_137, which does not adopt the salt-in strategy, displays a nonacidic proteome and an average isoelectric point of 8.10 (Fig. 4a; Table S3). In “*Ca*. Nanohalalkaliarchaeaceae,” NHA21 displays an isoelectric point profile similar to that of LC1Nh, one representative strain of “*Ca*. Nanosalinaceae” (Fig. 4a). Correspondingly, its average isoelectric point is 5.04, lower than those of most members of “*Ca*. Nanosalinaceae” (Table S3). Results of the *t* test indicate that the difference is significant (Text S1). Furthermore, the amino acid composition was compared. NHA21 has a high proportion of glutamate and aspartate in its proteome as *Halobacteria* and “*Ca*. Nanosalinaceae” (Fig. 4b; Table S3). These data support the idea that NHA21 employs the salt-in strategy.

10.1128/msystems.00669-22.3 FIG S3 Isoelectric point profile of other members in “*Ca*. Nanohaloarchaeota.” Horizontal coordinates are isoelectric points, and longitudinal coordinates are the frequencies of proteins in the proteomes at each isoelectric point. The isoelectric point of each protein is predicted based on the amino acid sequence. The isoelectric point profiles of bin width 0.1 are shown. Download FIG S3, TIF file, 1.1 MB. Copyright © 2022 Zhao et al. 2022 Zhao et al. https://creativecommons.org/licenses/by/4.0/ This content is distributed under the terms of the Creative Commons Attribution 4.0 International license.

10.1128/msystems.00669-22.8 TABLE S3 Average isoelectric points and amino acid compositions of the predicted proteomes of “*Ca*. Nanohaloarchaeota” and reference species. Download Table S3, XLSX file, 0.02 MB. Copyright © 2022 Zhao et al. 2022 Zhao et al. https://creativecommons.org/licenses/by/4.0/ This content is distributed under the terms of the Creative Commons Attribution 4.0 International license.

In “*Ca*. Nanoanaerosalinaceae,” the isoelectric point profiles are also acid shifted, but the degree is weak (Fig. 4a). Their average isoelectric points range from 5.69 to 6.16 (Table S3). *t* tests reveal that they are significantly higher than those of “*Ca*. Nanosalinaceae” but not significantly different from those of salt-in Salinibacter ruber and salt-out Spiribacter salinus (Text S1). However, they also contain a high proportion (more than 16%) of acidic amino acids (Fig. 4b; Table S3). It seems that “*Ca*. Nanoanaerosalinaceae” may maintain moderately acidic proteomes and moderate concentrations of intracellular inorganic salt.

**FIG 5:**
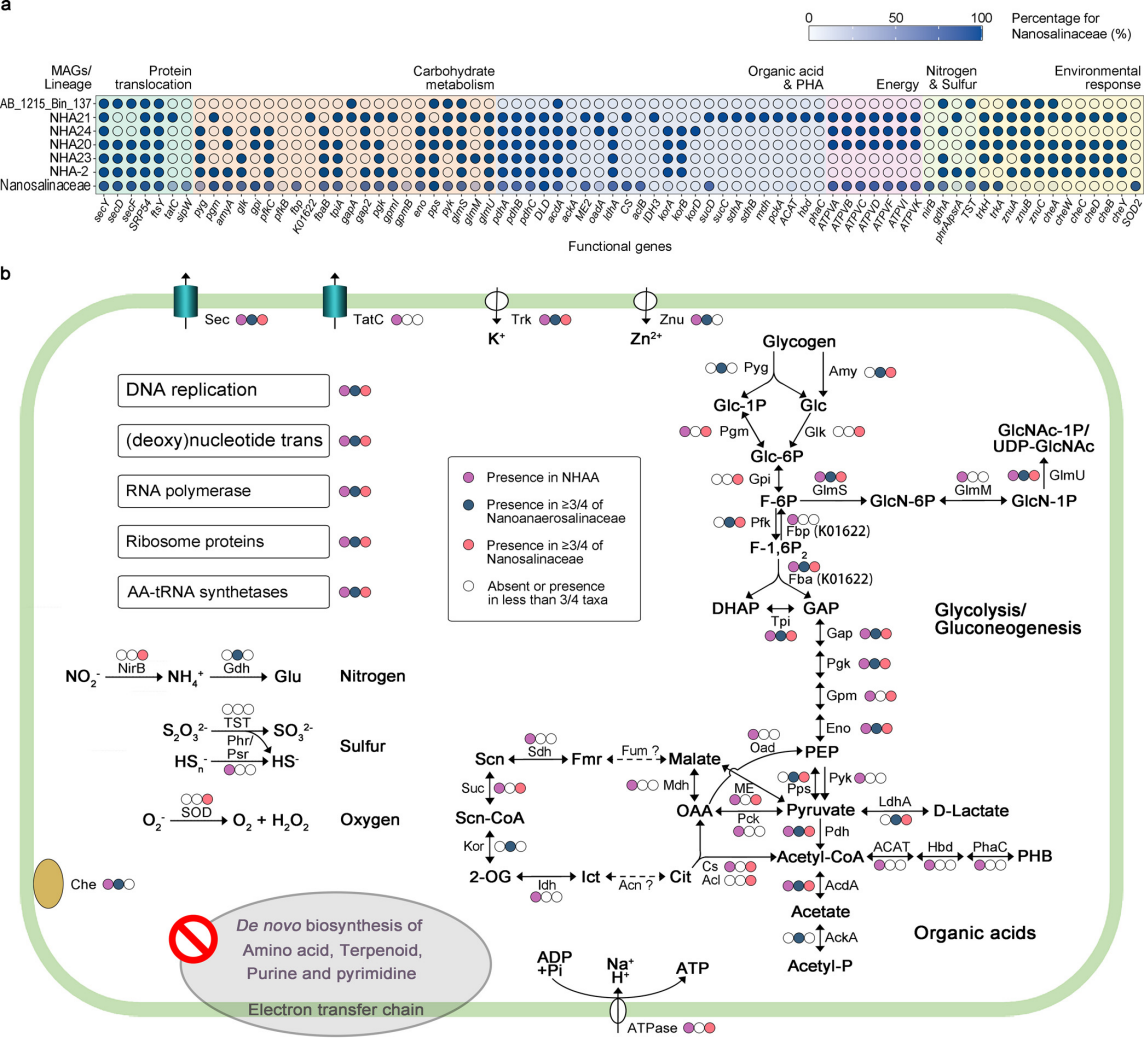
Functional potentials of the phylum “*Ca.* Nanohaloarchaeota” lineages. (a) Dot plot showing the presence or absence of genes involved in metabolism and environmental response in the members of the two novel families and the percentage in the family “*Ca*. Nanosalinaceae.” Solid and hollow dots indicated presence and absence in the genomes, respectively. Transparent blues show the percentage of genes in 14 “*Ca*. Nanosalinaceae” genomes. (b) Reconstruction of functional potentials in three families of “*Ca.* Nanohaloarchaeota.” The process was estimated based on the genes involved in genetic information processing, metabolism, and environmental stress response. Solid dots with different colors indicate the presence of the process or gene(s) in the three families, i.e., “*Ca*. Nanohalalkaliarchaeaceae” (NHAA) represented by NHA21, “*Ca.* Nanoanaerosalinaceae,” and “*Ca.* Nanosalinaceae,” while hollow dots indicate the absence of the process or gene(s). Glc, glucose; Glc-1P, glucose 1-phosphate; Glc-6P, glucose 6-phosphate; F-6P, fructose 6-phosphate; F-1,6P_2_, fructose 1,6-bisphosphate; DHAP, dihydroxyacetone phosphate; G-3P, glyceraldehyde 3-phosphate; PEP, phosphoenolpyruvate; Glu, glutamate; OAA, oxaloacetate; Cit, citrate; Ict, isocitrate; 2-OG, 2-oxoglutarate; Scn-CoA, succinyl-CoA; Scn, succinate; Fmr, fumarate; Che, chemotaxis; GlcN-6P, glucosamine 6-phosphate; GlcN-1P, glucosamine 1-phosphate; GlcNAc-1P, *N*-acetylglucosamine 1-phosphate; UDP-GlcNAc, UDP-*N*-acetylglucosamine; PHB, poly-hydroxybutyrate. Enzyme abbreviations are listed in Table S4.

In *Halobacteria* such as Haloarcula hispanica, Haloferax mediterranei, and Natronococcus occultus (representatives of the three orders), the mole percentages of glutamate and aspartate are higher than that in Escherichia coli, and levels of the two amino acids are almost equal. In “*Ca*. Nanosalinaceae” and “*Ca*. Nanoanaerosalinaceae” members, glutamate is unexpectedly much more abundant than aspartate, although both amino acid residues are also richer than in E. coli (Fig. 4b; Table S3). The result suggests that “*Ca*. Nanosalinaceae” and “*Ca*. Nanoanaerosalinaceae” achieve the salt-in strategy with more glutamate accumulation in the proteomes, and they are different from their *Halobacteria* hosts. We infer that most “*Ca*. Nanohaloarchaeota” lineages prefer glutamate, considering that AB_1215_Bin_137 contains about 12% acidic amino acids (close to nonhalophiles), and glutamate is likewise much more abundant than aspartate (Table S3); the characteristic of more glutamate is conserved in most “*Ca*. Nanohaloarchaeota.” More than half of the representative genomes have a glutamate dehydrogenase gene (*gdhA*) for glutamate biosynthesis (Fig. 5a; Table S4; described below); it might be a metabolic basis for the more glutamate. An exception is NHA21 of “*Ca*. Nanohalalkaliarchaeaceae,” which has almost equivalent levels of glutamate and aspartate in its proteome (Fig. 4b; Table S3). This suggests that “*Ca*. Nanohalalkaliarchaeaceae” may evolve along a different path from “*Ca*. Nanoanaerosalinaceae,” although they share a close phylogenetic relationship and the same habitat of hypersaline sediments. The two novel families may also employ the salt-in strategy to balance the osmotic pressure in hypersaline habitats.

### The novel families contain divergent potentials of metabolism and environmental response.

Cluster analysis based on the 3,007 orthogroups of “*Ca*. Nanohaloarchaeota” and the closely related phylum EX4484-52 reveals the functional difference among the three families (Fig. S4), so we compared their functional potentials. Generally, all genomes of “*Ca*. Nanohalalkaliarchaeaceae” and “*Ca*. Nanoanaerosalinaceae” have genes involved in the DNA replication apparatus, RNA polymerase complex, multiple ribosome proteins, and aminoacyl-tRNA biosynthesis, like those of “*Ca*. Nanosalinaceae” (Table S4). Meanwhile, they all lacked the complete genes for the electron transfer chain and the *de novo* biosynthesis of amino acids (except glutamate), purine, pyrimidine, and terpenoid for cell membranes (Fig. 5; Table S4). It is obvious that most “*Ca*. Nanohaloarchaeota” members have only draft genomes. Theoretically, the absence of a gene in a genome might be a false-negative result from the incompleteness of the genome. Nevertheless, the data above also support the idea that “*Ca*. Nanohaloarchaeota” members may have a symbiotic lifestyle. We found that the different genes were mainly involved in metabolism and environmental response, and then we estimated historical events (originations, duplications, transfers, and losses) by using the amalgamated likelihood estimation (ALE) approach (42, 43) and selecting the orthogroups that achieved a threshold of 0.3 in the raw reconciliation frequencies to avoid misses of true events (44).

10.1128/msystems.00669-22.4 FIG S4 Nonmetric multidimensional scaling (NMDS) analysis of functional gene profiles. A gene count matrix of 3,007 orthogroups in the 20 “*Ca*. Nanohaloarchaeota” and nine EX4484-52 genomes was used to compare the dissimilarity of functional profiles. The nonmetric fit (*R*^2^) between ordination distance and observed dissimilarity is equal to 0.978. Fourteen “*Ca*. Nanosalinaceae”, five “*Ca*. Nanoanaerosalinaceae”, nine EX4484-52 members, and AB_1215_Bin_137 are marked in red, green, grey, and blue, respectively. Download FIG S4, TIF file, 0.3 MB. Copyright © 2022 Zhao et al. 2022 Zhao et al. https://creativecommons.org/licenses/by/4.0/ This content is distributed under the terms of the Creative Commons Attribution 4.0 International license.

**FIG 6:**
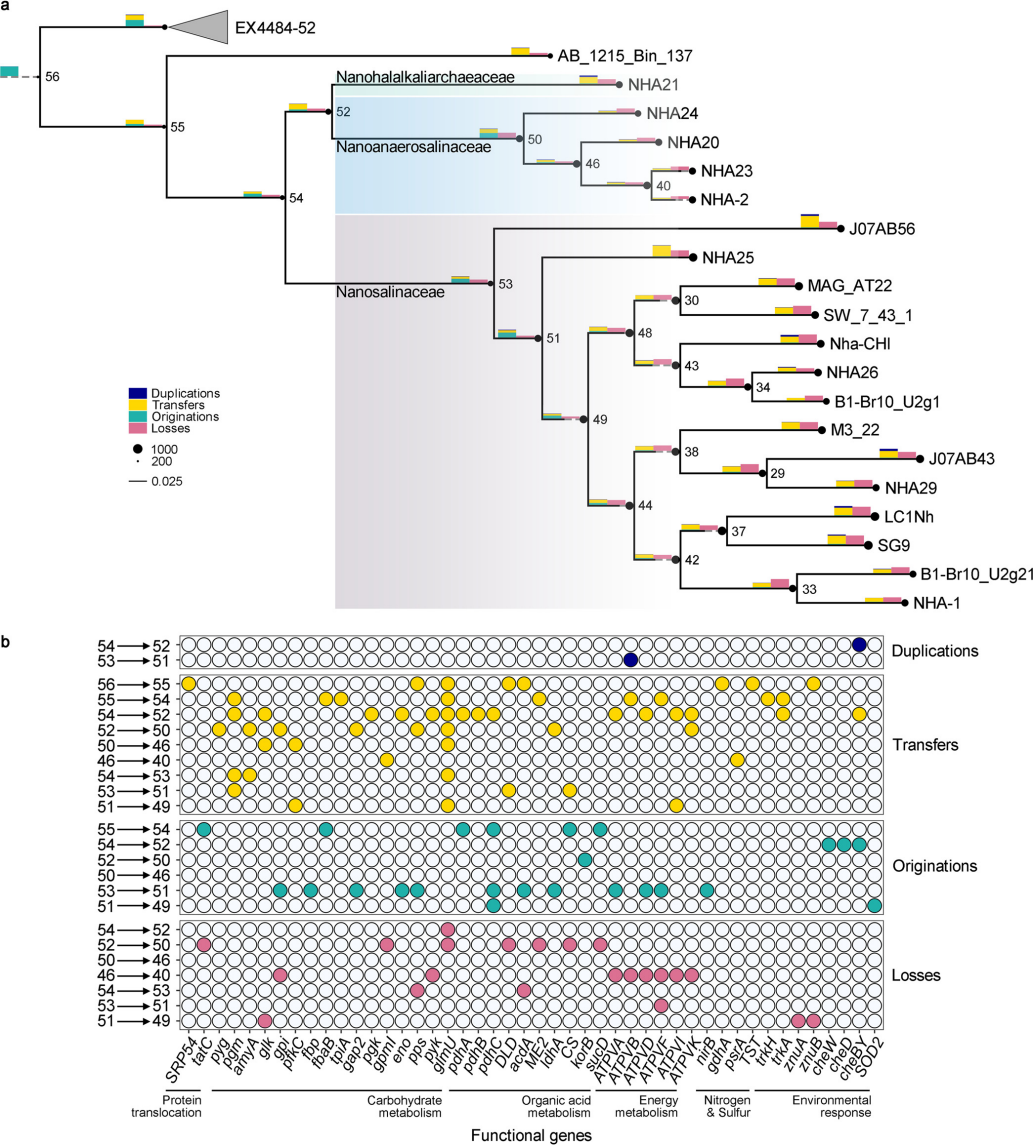
History event approximation in the phylum “*Ca*. Nanohaloarchaeota.” (a) Ancestral reconstruction tree of “*Ca*. Nanohaloarchaeota.” The consensus tree of ultrafast bootstrap approximation based on 124 single-copy ubiquitous orthogroups in representative genomes is exhibited. The historical events are approximated based on the species tree and 1,629 gene UFBOOT trees. The radii of the black circles at the nodes represent their (inferred) genome sizes. Some internal nodes of interest are marked by Arabic numerals. The bar charts above the horizontal branches represent the numbers of duplications, transfers, originations, and losses (bar heights in the legend correspond to 300 events each). Some branches were extended with dashed lines to fit the width of the bar charts. The branches leading to the phylum EX4484-52 were collapsed. (b) Dot plot showing the functional annotation of the historical event at the interest nodes. The orthogroups that achieved a threshold of 0.3 in the raw reconciliation frequencies are reported. The putative functions of orthogroups were estimated by medoid sequences, which have the highest sum of similarity scores with all other sequences based on the BLOSUM62 substitution matrix.

We first reconstructed glycolysis or gluconeogenesis and related pathways, which are reported to be involved in the symbiosis of the nanohaloarchaeon “*Candidatus* Nanohalobium constans” LC1Nh (18). However, many members of the families “*Ca*. Nanohalalkaliarchaeaceae” (represented by NHA21) and “*Ca*. Nanoanaerosalinaceae” lack some genes, including phosphoglycerate mutase genes (*gpmI* or *gpmB*), a glucose-6-phosphate isomerase gene (*gpi*), and some coding genes involved in alpha-glycan (such as glycogen) utilization (Fig. 5; Table S4). NHA21 does not have the ADP-dependent phosphofructokinase gene (*pfkC*) and pyruvate water dikinase gene (*pps*) but harbors a fructose-1,6-bisphosphatase (KEGG orthology identifier K01622) gene for gluconeogenesis and oxaloacetate decarboxylase (Na^+^ extruding; *oad*) for phosphoenolpyruvate (PEP) biosynthesis from pyruvate. Generally, the two novel families are not so proficient in carbohydrate metabolism as “*Ca*. Nanosalinaceae.” Therefore, we suppose that the carbohydrate metabolism may drive the development of the close association between “*Ca*. Nanosalinaceae” and *Halobacteria*. The observations support the idea that the genes *gpi*, *fbp*, *gap2*, *eno*, and *pps*, involved in glycolysis or gluconeogenesis, were estimated originations from node 53 to node 51 (Fig. 6a and b). These events occurred at the node of the last common ancestor (LCA) of most “*Ca*. Nanosalinaceae” members with the exception of J07AB56. Actually, the complete genome of J07AB56 is necessary to give a definite answer.

Obviously, NHA21 distinguishingly harbors many genes involved in organic acid conversion (including *IDH3*, *sucC*, *sdhAB*, *mdh*, and *pckA*) and polyhydroxybutyrate (PHB) biosynthesis (Fig. 5). Considering the incompleteness of the citrate cycle in NHA21, the organic acid metabolism-related genes may participate in energy production and reducing power balance. Similarly, we also found organic acid metabolism in the other two families. We found that in some “*Ca*. Nanosalinaceae” members, malate dehydrogenase (*ME2*), acetate coenzyme A (acetate-CoA) ligase (ADP forming) (*acdA*), and pyruvate dehydrogenase (*pdh*) genes are located together and even form a gene cluster (Fig. S5a). These genes coupled the metabolism of malate, pyruvate, and acetate. Generally, pyruvate is considered a key nutrient in hypersaline environments, and it can be excreted by some members of the *Halobacteria* fed with glycerol (45). Meanwhile, glycerol is an important osmotic stabilizer produced by *Dunaliella*, a primary producer in hypersaline ecosystems (46). Therefore, pyruvate might be consumed with acetate and malate as products. In this process, ATP was generated, and reducing power was balanced (Fig. S5b). In “*Ca*. Nanoanaerosalinaceae,” the *ME2* gene is lacking, and other genes are not linked (Table S4). However, they commonly have lactate dehydrogenase gene (*ldhA*), whose protein product could take on the role of ME2 in NAD^+^ regeneration (Fig. S5c). Overall, there are metabolic variances of organic acids among the three families (Fig. 5). We found that organic acid metabolism-related genes show complex evolutionary events (Fig. 6), and this might lead to the metabolic diversity of organic acids.

10.1128/msystems.00669-22.5 FIG S5 Gene arrangement of *ME*, *acdA*, *pdhABC*, and *DLD* in the “*Ca*. Nanohaloarchaeota” lineages. (a) Longitudinal coordinate exhibited the lineage and contig names. The genes were aligned by the *pdhA* gene. The number in the horizontal coordinate indicates the position in the contig, and a minus signs indicates that the contig sequence is a reverse complement. The length and direction of the arrow show the gene length and direction. The enzyme abbreviations are listed in Table S4. (b and c) Relevant biochemical reactions. Download FIG S5, TIF file, 0.6 MB. Copyright © 2022 Zhao et al. 2022 Zhao et al. https://creativecommons.org/licenses/by/4.0/ This content is distributed under the terms of the Creative Commons Attribution 4.0 International license.

In addition to metabolism, some environmental responses are different. The subunits SecYDF of the Sec-dependent protein export system are present in most members of all three families, while the TatC subunit of the twin-arginine translocation system is present in “*Ca*. Nanohalalkaliarchaeaceae” (represented by NHA21) as well as some members of the family “*Ca*. Nanosalinaceae” but not in “*Ca*. Nanoanaerosalinaceae” members (Fig. 5a; Table S4). The Tat system exports the folded protein, and this process can avoid protein denaturation during the unfolding and refolding under hypersaline conditions (47). Consistently, TatC is found to exist in “*Ca*. Nanohalalkaliarchaeaceae” and some “*Ca*. Nanosalinaceae” with a more acidic proteome (implying high intracellular salinity [see above]). *tatC* was an estimated origination from node 55 to node 54, and then it might be lost from node 52 to node 50 (Fig. 6a and b). The evolutionary analysis backs the functional prediction.

Furthermore, we observed that chemotaxis-related genes (*cheAWCDBY*) and zinc transporter genes (*znuABC*) are present in “*Ca*. Nanohalalkaliarchaeaceae” and “*Ca*. Nanoanaerosalinaceae,” while they are not in any of the 14 representative genomes of “*Ca*. Nanosalinaceae” (Fig. 5a). It was reported that zinc was important for bacterial chemotaxis (48), and it may play a similar role in the novel archaeal lineages. Both “*Ca*. Nanohalalkaliarchaeaceae” and “*Ca*. Nanoanaerosalinaceae” survive in hypersaline sediment (Fig. 2), and the sediment is not so inhabitable for *Halobacteria* as brine (29, 36). The chemotaxis is possibly significant in seeding out the *Halobacteria* hosts. Conversely, an Fe-Mn family superoxide dismutase gene (*SOD2*) is annotated in the family “*Ca*. Nanosalinaceae” but not in “*Ca*. Nanoanaerosalinaceae” (Fig. 5; Table S4). Accordingly, superoxide dismutase may play a part in response to the reactive oxygen species superoxide in aerobic environments. The evolutionary result predicted that the chemotaxis-related genes *cheWDY* were originations from node 54 to node 52, while the *SOD2* gene was an origination from node 51 to node 49 (Fig. 6a and b).

### Deduction of the symbiotic evolution of “*Ca*. Nanohaloarchaeota” with *Halobacteria*.

The two novel families are located at the root of the “*Ca*. Nanosalinaceae.” Meanwhile, they exhibit differences in habitat distribution, close connection with *Halobacteria* (inferred from HGTs from *Halobacteria*), proteome acidification, and functional potentials (described above). Most DPANN lineages inhabit marine or low-salt environments (9–11, 14, 49). Therefore, it is reasonable that the adaptational evolution into extreme hypersaline habitats occurs in “*Ca*. Nanosalinales.” Following this idea, we consider that the acidification of predicted proteomes of members in “*Ca*. Nanohaloarchaeota” gradually took place during this process. “*Ca*. Nanosalinaceae” represents a developing direction of adaptation to extreme hypersaline environments. In fact, the two novel families are also the offspring inhabiting modern environments, and they maintain certain acidifications of proteomes from their LCAs. Therefore, we emphasize that, in other words, the two novel families are not the intermediate status itself but may inherit similar characteristics.

All three families were found in hypersaline habitats, i.e., brine, surface sediment, and deep sediment (Fig. 2). They are selected by different ecological niches, but they could be used to analyze phylogenetic and evolutionary relationships. One of the reasons is that “*Ca*. Nanosalinaceae” members resist the oxygen in the brine, but they cannot perform aerobic respiration as well as the two novel families (Fig. 5; Table S4). In addition, as a member of “*Ca*. Nanosalinaceae,” NHA25 seems to prefer anaerobic environments (Table S2), and it is the only one that does not have the *SOD2* gene (Table S4). Possibly, the absence of this gene restricts NHA25 in anaerobic environments. In other words, habitat preference for brine and sediment may depend on a few genes. In addition, the ecological niches of the haloarchaeal hosts should also be considered; i.e., they thrive in both brine and sediment. Some *Halobacteria* are able to perform both aerobic respiration and anaerobic respiration (39, 40), and they could exist as hosts for these families. In addition, it is also possible that the three “*Ca*. Nanohaloarchaeota” families might live with different haloarchaeal lineages, and their common ancestor might have first established a physical connection with one *Halobacteria* ancestor, or they may share a molecular apparatus for interaction. Further evolutionary analysis on *Halobacteria* or cultivation-based research may give a definite answer to the question. In any case, however, *Halobacteria* lineages could be regarded as the common hosts for the evolution of the three “*Ca*. Nanohaloarchaeota” families.

Based on this research, we propose that the development of a close association between “*Ca*. Nanosalinaceae” and their *Halobacteria* hosts might be derived from the weaker connection like “*Ca*. Nanoanaerosalinaceae.” Similar to hypersaline adaptation, “*Ca*. Nanoanaerosalinaceae” may maintain the weak interaction with *Halobacteria*, while “*Ca*. Nanosalinaceae” make an enhancement. In addition, “*Ca*. Nanohalalkaliarchaeaceae,” represented by NHA21, also show an close association like “*Ca*. Nanosalinaceae.” NHA21 was most likely to establish an association with the order “*Candidatus* Halarchaeoplasmatales” (the new name for “*Candidatus* Haloplasmatales” [29]), because the order was reported to be the most abundant archaeal lineage in some hypersaline deep sediments (29, 36, 50). Nevertheless, they also form a close relationship with *Halobacteria* (Fig. 3). This might be the result of evolution from the weak connection represented by “*Ca*. Nanoanaerosalinaceae.” Apparently, “*Ca*. Nanohalalkaliarchaeaceae” has an evolutionary path different from that of “*Ca*. Nanosalinaceae” (Fig. 6). The convergent evolution to close connection indicates the advantage of “*Ca*. Nanohaloarchaeota” symbiosis with *Halobacteria*. In conclusion, the comparative genomic analysis of the novel lineages provides evidence of symbiotic evolution, although the metabolic prediction and evolutionary approximation could be further improved by relying on the complete genomes in the future. Moreover, the coculture of the novel families with *Halobacteria* would possibly offer supplementary insights.

### Conclusion.

We report two novel families, “*Ca*. Nanoanaerosalinaceae” and “*Ca*. Nanohalalkaliarchaeaceae.” The two families prefer the habitat hypersaline sediment and are placed at the root of the phylogenetic trees of “*Ca*. Nanosalinaceae.” In addition, “*Ca*. Nanoanaerosalinaceae” contain a lower proportion of genes horizontally acquired from *Halobacteria*, while both novel families exhibit distinct proteomic characteristics for hypersaline adaptation and different functional potentials, including carbohydrate metabolism, organic acid metabolism, chemotaxis, reactive oxygen species response (*SOD2*), and hypersaline adaptation (Tat protein translocation system). The two novel families broaden the archaeal diversity of “*Ca*. Nanohaloarchaeota” and provide insights into symbiotic evolution. This research will also provide a model for similar studies in other DPANN lineages.

### Description of the family “*Ca*. Nanohalalkaliarchaeaceae” and taxa classified in the family.

“*Ca*. Nanohalalkaliarchaeaceae” fam. nov. (Na.no.hal.al.ka.li.ar.chae.a.ce’ae, N.L. fem. n. *Nanohalalkaliarchaeum*, Candidatus generic name; -*aceae*, designating a family; N.L. fem. pl. n. *Nanohalalkaliarchaeaceae*, the *Nanohalalkaliarchaeum* family). The type genus of the family is “*Candidatus* Nanohalalkaliarchaeum.”

“*Ca*. Nanohalalkaliarchaeum” gen. nov. (Na.no.hal.al.ka.li.ar.chae’um. Gr. masc. n. *nanos*, a dwarf; Gr. masc. n. *hals* (*gen. halos*), salt of the sea; N.L. neut. n. *alkali*, from Arabic *al*, “the,” and Arabic n. *qaliy*, ashes of saltwort; N.L. neut. n. *archaeum*, archaeon; from Gr. adj. *archaîos -a -on*, ancient; N.L. neut. n. *Nanohalalkaliarchaeum*, a dwarf haloalkaliphilic archaeon). The type species of the genus is “*Candidatus* Nanohalalkaliarchaeum halalkaliphilum.”

“*Ca*. Nanohalalkaliarchaeum halalkaliphilum” sp. nov. (hal.al.ka.li’phi.lum. Gr. masc. n. *hals* (*gen*. *halos*), salt; N.L. neut. n. *alkali*, alkali; N.L. adj. *philus -a -um*, friend, loving; from Gr. adj. *philos -ê -on*, loving; N.L. neut. adj. *halalkaliphilum*, salt and alkali loving). The type material is the metagenome-assembled genome NHA21, whose GenBank accession number (whole genome sequencing [WGS]) is JALDAF000000000.

### Description of the family “*Ca*. Nanoanaerosalinaceae” and taxa classified in the family.

Description of “*Ca*. Nanoanaerosalinaceae” fam. nov. (Na.no.an.ae.ro.sa.li.na.ce’ae, N.L. fem. n. *Nanoanaerosalina*, Candidatus generic name; -*aceae*, designating a family; N.L. fem. pl. n. *Nanoanaerosalinaceae*, the *Nanoanaerosalina* family). The type genus of the family is the genus “*Candidatus* Nanoanaerosalina.”

Description of “*Ca*. Nanoanaerosalina” gen. nov. (Na.no.an.ae.ro.sa.li’na. Gr. masc. n. *nanos*, a dwarf; Gr. pref. *an*-, not; Gr. masc. n. *aêr* (*gen*. *aeros*), air; N.L. masc. adj. *salinus*, saline; N.L. fem. n. *Nanoanaerosalina*, a dwarf saline organism not living in air). The type species of the genus is “*Candidatus* Nanoanaerosalina halalkaliphila.”

Description of “*Ca*. Nanoanaerosalina halalkaliphila” sp. nov. (hal.al.ka.li’phi.la. Gr. masc. n. *hals*, (*gen*. *halos*), salt; N.L. neut. n. *alkali*, alkali; N.L. adj. *philus -a -um*, friend, loving; from Gr. adj. *philos -ê -on*, loving; N.L. fem. adj. *halalkaliphila*, salt and alkali loving). The type material is the metagenome-assembled genome NHA20, whose GenBank accession number (WGS) is JALDAE000000000.

## MATERIALS AND METHODS

### Enrichment culture and metagenomic sequencing.

Deep sediment samples from five crystallizer ponds of different salinities (1, 3, 15, 24, and 33%) described in the previous study (29) were used for enrichment culture. In brief, 5 g of four mixed sediment samples (from the identical pond) was added with 5 mL of corresponding brine and 5 mL sterile medium with the same salinity as the brine. The five media were composed of (per liter) 0.2 mg MgCl_2_·6H_2_O, 0.05 g KH_2_PO_4_, 2 g KCl, and variable concentrations of NaCl, NaHCO_3_, and CaCl_2_ for samples from different ponds. Their concentrations (per litre) were 10 g, 2 g, and 1.2 mg, respectively, for the 1% samples; 30 g, 3 g, and 0.4 mg for 3%; 150 g, 13 g, and 6.5 mg for 15%; 240 g, 28 g, and 7.7 mg for 24%; and 370 g, 5 g, and 4.1 mg for 33%. After sterilization, pH values were usually approximately 10.0. After premixing the samples with brine and media, a final concentration of 2.42 g/L Na_2_MoO_4_·2H_2_O, 5 mg/L kanamycin, 20 mg/L ampicillin, 2.4 g/L NaS_2_·9H_2_O, and either 0.52 g/L sodium formate dihydrate (indicated with “F” in sample names), 0.68 g/L sodium acetate trihydrate (indicated with “A”), or no substrate was added (details shown in Table S5 [https://doi.org/10.6084/m9.figshare.19549495]). A total of 15 samples were anaerobically and statically incubated at 30°C without light for about 210 days. Then, microbial cells with insoluble matter were collected using centrifugation at 4°C. Total DNA was extracted from the samples using a PowerSoil DNA isolation kit (MoBio, CA, United States) for metagenome sequencing. However, there was not enough DNA from the sample of 1% salinity with no substrate. Therefore, 14 competent DNA samples were used for subsequent library construction and metagenomic sequencing in the Illumina platform HiSeq X 10 to generate 150-bp paired-end reads (Table S5 [https://doi.org/10.6084/m9.figshare.19549495]).

### Contig assembly and genome binning.

Quality control of raw reads of each metagenome was performed using the read_qc module with default parameters of the metaWRAP v1.2.2 pipeline (51). Clean reads generated for each sample were individually assembled into contigs using the assembly module of metaWRAP with the default assembler MEGAHIT v1.1.3 (52), and short contigs (<1,000 bp) were removed. Three different metagenomic tools for genome binning, CONCOCT v1.0.0 (53), MetaBAT2 v2.12.1 (54), and MaxBin2 v2.2.6 (55), integrated into the binning module of metaWRAP, were used to recover initial MAGs from each metagenome. In addition, to obtain more “*Ca*. Nanohaloarchaeota” MAGs, 18 metagenomes of brine and surface sediment samples from our previous study (24) were reanalyzed by following the genome-binning approach described below. Then, all MAGs obtained from the same metagenome were individually refined with a minimum completeness of 50% or 30% and a maximum contamination of 10% or 20% using the bin_refinement module in metaWRAP. Subsequently, the best representative genomes were chosen from all the refined MAGs using dRep v3.2.0 with a threshold of 99% ANI (56). Ten more “*Ca*. Nanohaloarchaeota” MAGs were obtained in this study (Table S6 [https://doi.org/10.6084/m9.figshare.19549495]).

### Genome collection, quality estimation, and function prediction.

The genome sequences of “*Ca*. Nanohaloarchaeota” and its closely related lineages, including the phyla “*Ca*. Aenigmarchaeota,” EX4484-52, PWEA01, and QMZS01, were collected (Table S1) by 9 October 2021. First, 232 genomes were downloaded from GenBank of the National Center for Biotechnology Information (NCBI; https://www.ncbi.nlm.nih.gov/) under the following taxonomy IDs: 743724 for the phylum “*Ca*. Aenigmarchaeota,” 1462430 for the phylum “*Ca*. Nanohaloarchaeota” in the DPANN group, 1051663 for the class “Nanohaloarchaea” in the phylum *Euryarchaeota*, and 2565780 for the unclassified DPANN group. Two genomes of “*Candidatus* Nanohaloarchaeum antarcticus” (17) were obtained via the identification numbers 2643221421 and 2791354821 in the Integrated Microbial Genomes (IMG) database (https://img.jgi.doe.gov/). Additionally, 148 archaeal genomes from our previous studies (24, 29) were also used. In addition, 2,285 archaeal representative genomes in GTDB were downloaded (Table S7 [https://doi.org/10.6084/m9.figshare.19549495]) for phylogeny analysis. The relative abundance of MAGs was estimated individually by mapping clean reads to MAGs with the genome module in CoverM v.0.6.0 (https://github.com/wwood/CoverM) using the following parameters: –min-read-percent-identity, 0.95; –min-read-aligned-percent, 0.75.

Classification was generally preanalyzed using the classify workflow in GTDB-Tk (V1.7.0) (57) based on release 202 in the Genome Taxonomy Database (https://gtdb.ecogenomic.org/). Subsequently, basic statistics (including contig number, genome size, *N*_50_, *N*_90_, and G+C content) of genomes were generated using the bbstats.sh script (last modified 25 July 2019) in the BBTools suite (sourceforge.net/projects/bbmap/). The genomes were annotated using Prokka (version 1.13) with the settings Archaea for annotation mode and RNAmmer (v1.2) for rRNA prediction (58, 59). In this process, Prodigal (v2.6.3) was used to find protein-coding features (60). The numbers of genes, coding sequences (CDSs), rRNAs, and tRNAs were retrieved from the Prokka output. Genome completeness and contamination were estimated using the lineage-specific workflow in CheckM (v1.1.3) (61). The isoelectric point of each protein was predicted using a protein isoelectric point calculator (62). Functions of CDSs in each genome were predicted using the Diamond method in eggNOG-mapper (v2.0.0) (63, 64). From the output, Clusters of Orthologous Genes (COG) categories (65) and Kyoto Encyclopedia of Genes and Genomes (KEGG) orthology (KO) identifiers were retrieved. Metabolic pathways were reconstructed using the online tool KEGG Mapper with KO annotation (https://www.genome.jp/kegg/). Carbohydrate-active enzymes were annotated using dbCAN2 (v2.0.6) based on the CAZy database version CAZyDB.07312019 (66, 67).

### Phylogeny and sequence identity analyses.

The phylogenetic trees were based on 122 archaeal ubiquitous single-copy proteins, ribosome proteins, and 16S rRNA genes with 2,285 archaeal genomes except NATU lineages in GTDB as an outgroup. Briefly, the multiple-sequence-alignment file of the 122 archaeal ubiquitous single-copy proteins were also produced using the classify workflow in GTDB-Tk (57). The consensus tree of ultrafast bootstrap approximation was reconstructed using IQ-TREE (multicore version 1.6.12) with an ultrafast bootstrap of 1,000 and standard model selection followed by tree inference (68). The best-fit model was chosen according to the Bayesian information criterion. The archaeal ribosome proteins were inferred from the genomes and then aligned, masked, and trimmed using AMPHORA2 with default options (69). The aligned and trimmed amino acid sequences of Rpl2p, Rpl3p, Rpl4lp, Rpl5p, Rpl6p, Rpl14p, Rpl18p, Rpl22p, Rpl24p, Rps3p, Rps8p, Rps10p, Rps17p, and Rps19p were concatenated using AMAS (70). The phylogenomic tree was reconstructed using IQ-TREE with the same settings, and the best-fit model was chosen. The archaeal 16S rRNA gene sequences were predicted using RNAmmer (v1.2) (59). Those 1,450 to 1,700 bp in length and of high quality (score above 1,100 and no ambiguous sites) were selected (Table S8 [https://doi.org/10.6084/m9.figshare.19549495]), referring to previous literature (59, 71). The sequences were aligned using MUSCLE v3.8.31 with default options (72). The phylogenetic reconstruction was also done using the same method, and the best-fit model was chosen. The phylogenetic trees were visualized using the Interactive Tree of Life (iTOL, version 6.5) (73). The archaeal trees were generally reconstructed by setting the root at the node between the DPANN group and other lineages. The phylogenetic trees of the NATU clade were reconstructed by following the same approach.

The identity between two 16S rRNA gene sequences was estimated using Nucleotide-Nucleotide BLAST 2.6.0+ (74). AAI between two sets of predicted coding sequences from the genomes (referred to as the predicted proteome here) was calculated using the online AAI calculator (http://enve-omics.ce.gatech.edu/aai/index). ANI between two genomes was computed using FastANI (version 1.33) with default options (75).

### Horizontal gene transfer event prediction.

The putative HGT events were computed using the HGTector pipeline version 2.0b3 (34). First, the GTDB taxonomy-based database for HGTector analysis was built by considering that different genomes of “*Ca*. Nanohaloarchaeota” were assigned to *Euryarchaeota* or the DPANN group. In brief, the taxonomy file was downloaded from GTDB within release 202, and then it was reformatted into NCBI taxdump style using a Python 3 script named gtdb_to_taxdump.py provided by HGTector contributors (https://github.com/qiyunlab/HGTector). Three files named names.dmp, nodes.dmp, and taxid.map were produced. All proteins of 2,339 archaeal representatives in GTDB were directly downloaded from GenBank or predicted based on the genomes using Prodigal (v2.6.3) (60). A GTDB-based and local prot.accession2taxid file was created based on the produced taxid.map file and all proteins. The database was built from all the proteins and the GTDB-based taxonomy files using the makedb command in Diamond v0.9.26.127 (64). After that, a batch homology search was performed using a Diamond method for each proteome. HGT events were predicted using the analyze command with the following settings: “self” group, “*Ca*. Nanohaloarchaeota” (whose taxid in the local GTDB-based taxonomy was 18); “close” group, “*Ca*. Nanohaloarchaeota” and EX8848-52 (taxid, 20); maximum number of hits, 12; maximum E-value cutoff, 1e−8; minimum percent identity cutoff, 30%; minimum percent query coverage cutoff, 50%; bandwidth for Gaussian KDE, auto. Donor’s taxonomy of HGT-derived genes was deciphered using the lineage command in TaxonKit (v0.9.0) (76).

### Comparative genomic analysis for ancestral reconstruction.

The comparative genomic analysis was performed by referring to the published research (44, 77, 78). Briefly, 3,007 orthogroups were found from 32,540 CDSs in the genome set (20 “*Ca*. Nanohaloarchaeota” and 9 EX4484-52 genomes) using OrthoFinder version 2.4.0 with default settings (79). The gene count matrix of orthogroups in the genomes was used to compare the functional profile. Nonmetric multidimensional scaling (NMDS) analysis was performed using the metaMDS function with default options in R package vegan v2.5-7 (https://github.com/vegandevs/vegan/).

We retained 1,629 orthogroups with 4 or more genes according to the principles in previous literature (77). The genes of identical orthogroup were aligned using MAFFT v7.407 with the L-INS-i method, which has high accuracy (80). The columns were removed using the heuristic automated1 method of trimAl version 1.2rev59 (81), and then the sequences containing too many gaps were abandoned with the following options: minimum overlap of a position with other positions, 0.3; minimum percentage of the satisfied positions, 50. For the reconstruction of the species tree, 124 single-copy orthogroups were manually selected that were present in no fewer than 18 “*Ca*. Nanohaloarchaeota” genomes (≥90%) and no fewer than 5 EX4484-52 genomes (>50%). The aligned and trimmed sequences were concatenated using AMAS (70). Correspondingly, the phylogenomic tree was reconstructed using IQ-TREE, and the best-fit model of LG+F+I+G4 was chosen. The root of this species tree was reset using the midpoint method, a built-in function in iTOL (73). To obtain the UFBOOT trees of the 1,629 orthogroups, we used IQ-TREE with the same settings (-m, LG+G; -bb, 1000; -wbtl) as in the previous research (77). The frequencies of duplications, transfers (gene transfers from the lineage inside the species tree), losses, and originations (gene transfers from the lineages outside the species tree, or true gene originations), as well as copy numbers of the 1,629 orthogroups at each node, were inferred using maximum-likelihood estimation (ALEml_undated command) in ALE v0.4 (42). The number of each event and genome size were inferred by parsing the .uml_rec files of the ALE output using the Python scripts set named ALE helper (78). The details of orthogroups and the output files can be accessed via the link in “Data availability.” The ancestral reconstruction tree was visualized using ETE Toolkit version 3.1.2 (82).

The orthogroups that achieved a threshold of 0.3 in the raw reconciliation frequencies were counted. This threshold is relaxed but necessary to avoid missing many true events (44). A medoid sequence was selected from each orthogroup as the representative for functional annotation. The medoid sequences have the highest sum of similarity scores with all other sequences based on the BLOSUM62 substitution matrix using Protein-Protein BLAST 2.6.0+ (74).

### Statistical analysis.

Student’s *t* test or the Wilcoxon test was performed using the functions in the library ggpubr to compare the means between any two families’ ApI, the ratio of HGTs from *Halobacteria*, and G+C content. Briefly, the normal distribution of data was first tested to decide between the parametric *t* test and the nonparametric Wilcoxon test. The comparisons between NHA21 (representing “*Ca*. Nanohalalkaliarchaeaceae”) and the other two families were performed using a one-sample test, while the comparisons between “*Ca*. Nanoanaerosalinaceae” and “*Ca*. Nanosalinaceae” were performed using an unpaired two-sample test.

### Data availability.

The “*Ca*. Nanohaloarchaeota” genomes are available from the NCBI under the BioProject identifier PRJNA797678. DNA sequencing data have been deposited in BioProject with the identifiers PRJNA549802 and PRJNA679647. Metagenomic sequencing data of 14 enrichment samples are deposited in BioProject with the identifier PRJNA769545. Raw data (including protein files, tree files, horizontal gene transfer analysis, comparative genomics files, etc.) generated in this study, Fig. S6 to S10, and Table S5 to S9 are available at https://doi.org/10.6084/m9.figshare.19549495.
