# Supplementary figures and images for: Comparative Genomic Insights into the Evolution of Halobacteria-Associated “Candidatus Nanohaloarchaeota”
Source: mSystems. 2022 Oct 19;7(6):e00669-22. doi: 10.1128/msystems.00669-22 (PMC9765267; doi:10.1128/msystems.00669-22)

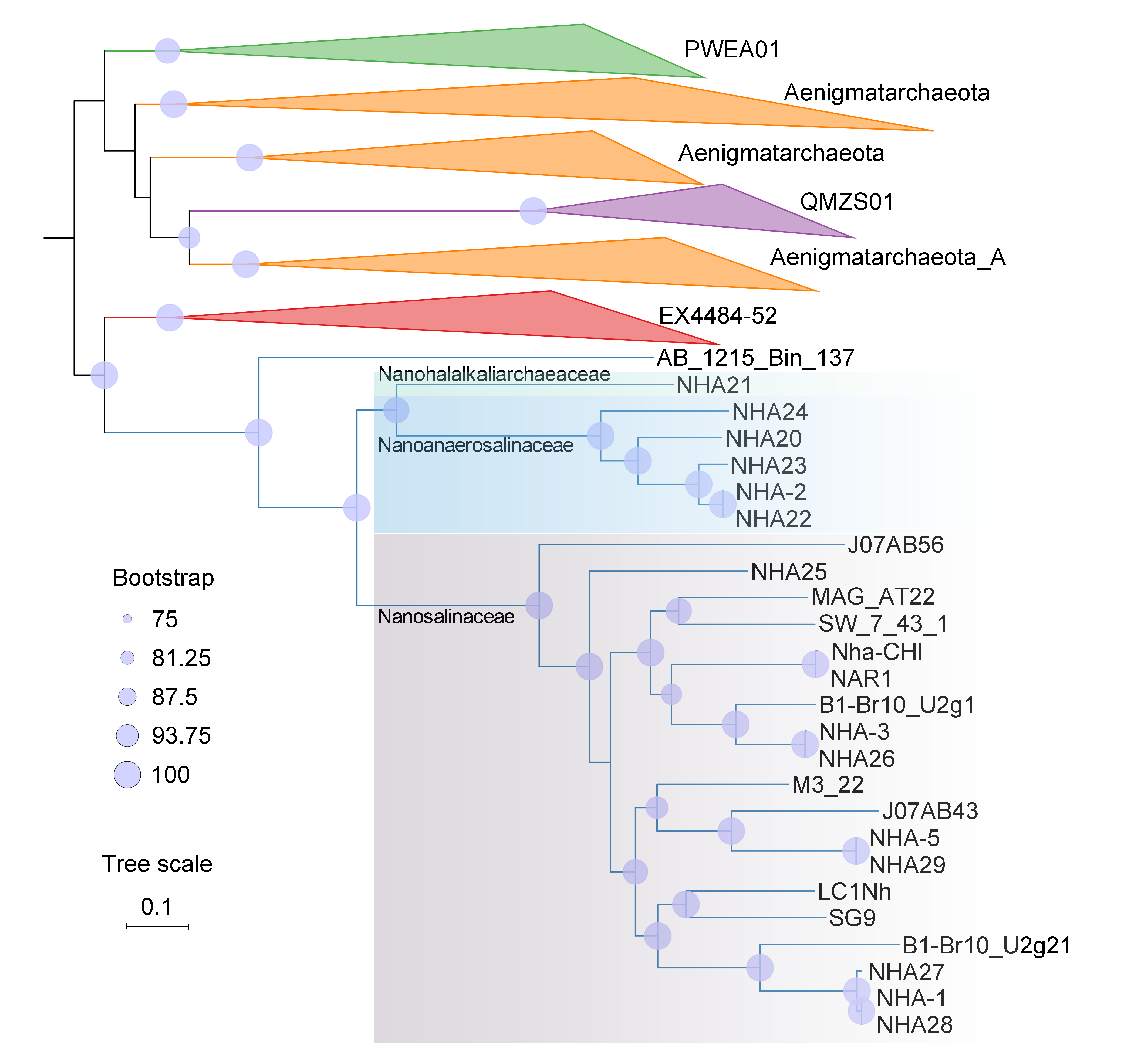

Supplement: FIG S1 [file msystems.00669-22-s0002.tif]

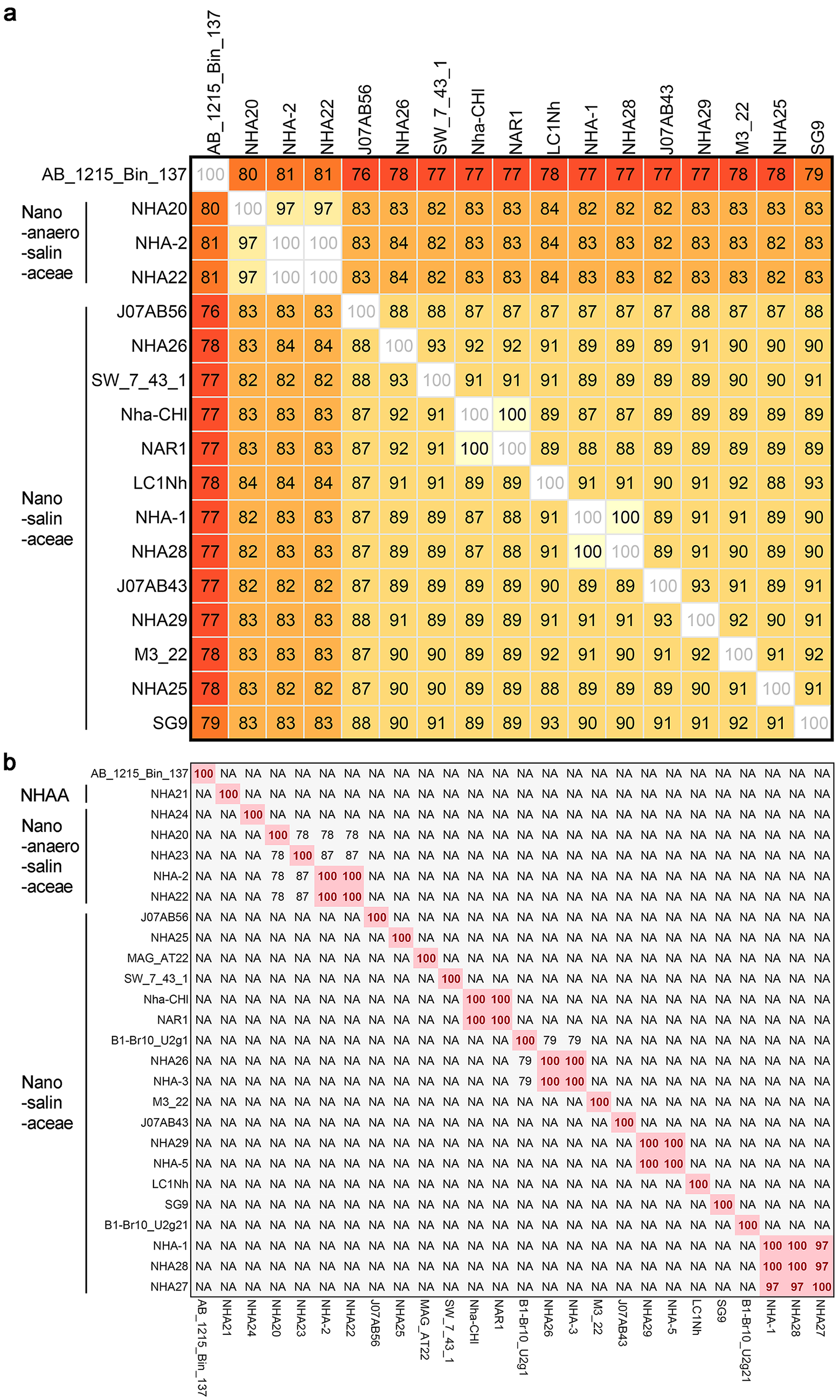

Supplement: FIG S2 [file msystems.00669-22-s0003.tif]

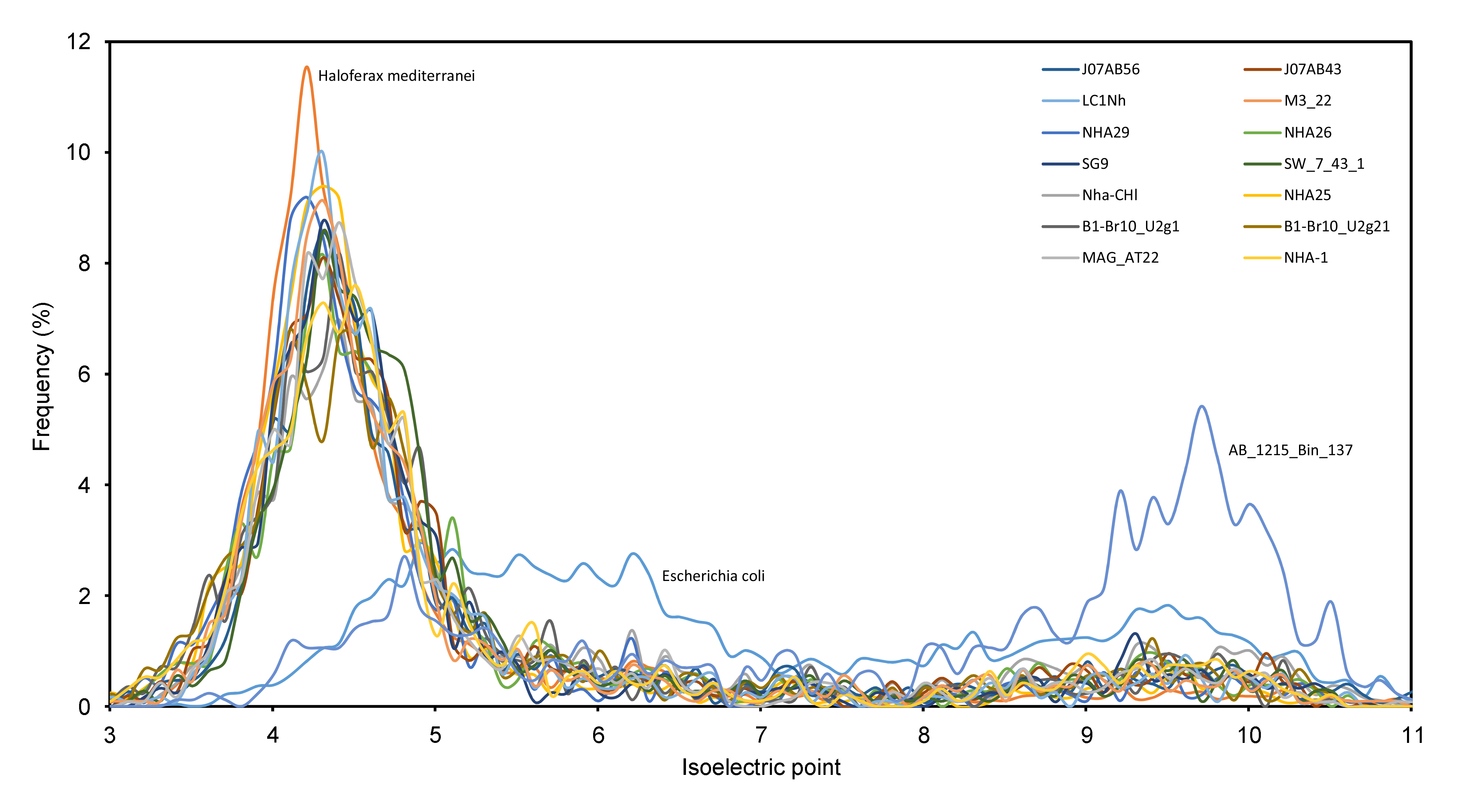

Supplement: FIG S3 [file msystems.00669-22-s0004.tif]

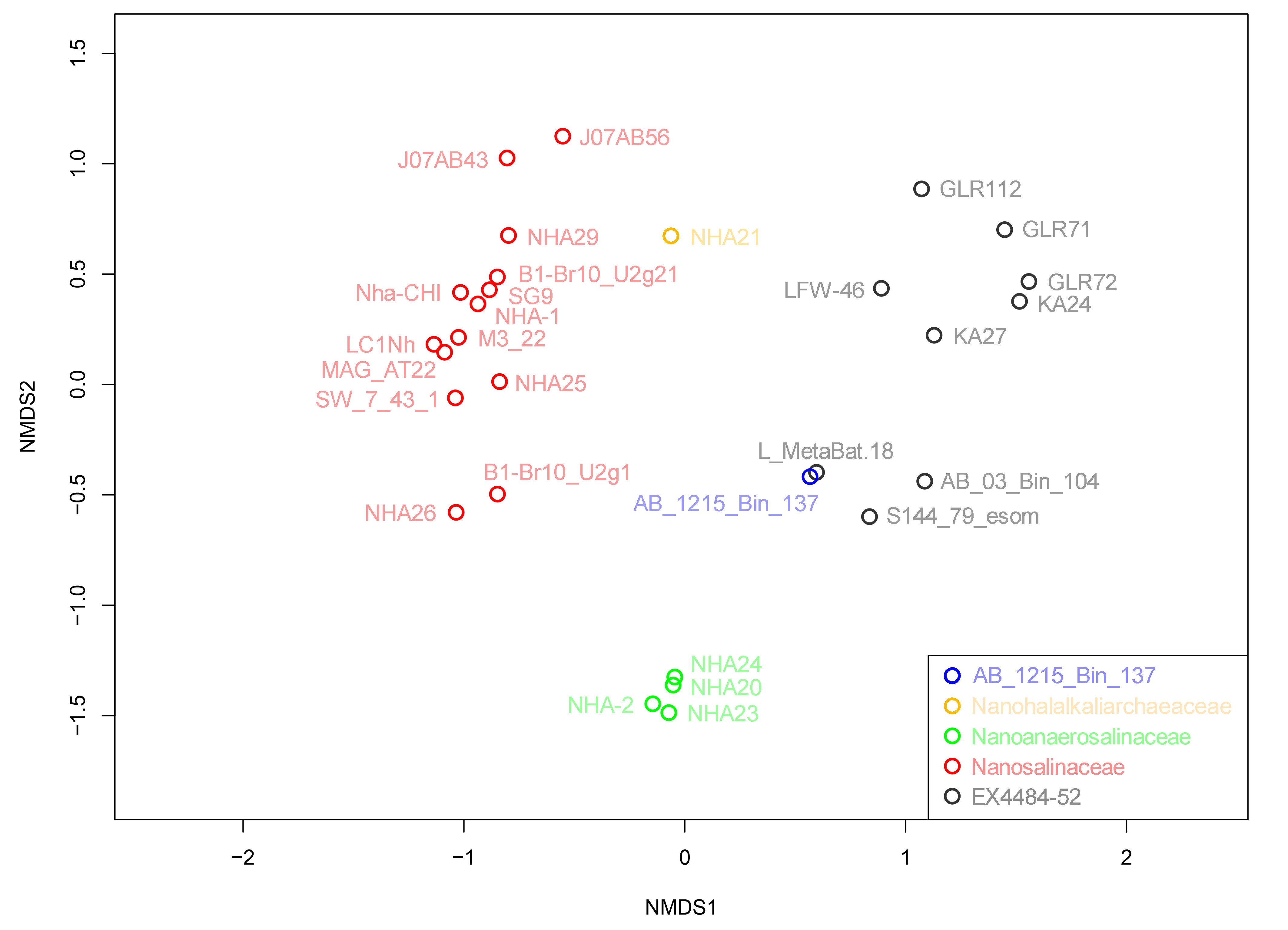

Supplement: FIG S4 [file msystems.00669-22-s0005.tif]

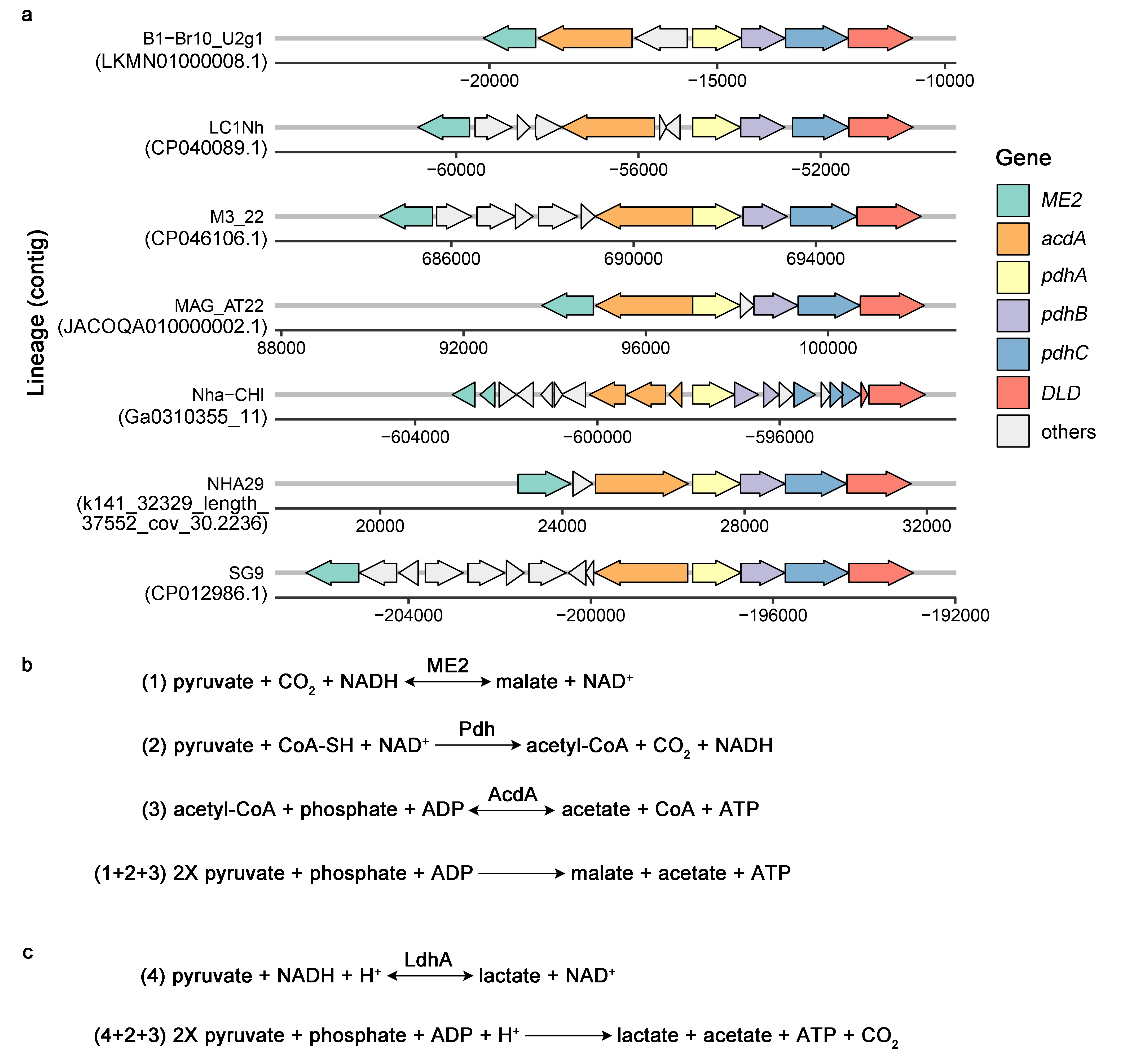

Supplement: FIG S5 [file msystems.00669-22-s0006.tif]
