# Supplementary material for: Comparative Genomic Insights into the Evolution of Halobacteria-Associated “Candidatus Nanohaloarchaeota”
Source: mSystems. 2022 Oct 19;7(6):e00669-22. doi: 10.1128/msystems.00669-22 (PMC9765267; doi:10.1128/msystems.00669-22)
Supplement: TEXT S1 [file msystems.00669-22-s0001.pdf]

Supplementary Results

Comparative genomic insights into the evolution of *Halobacteria*-associated "*Candidatus* Nanohaloarchaeota"

Dahe Zhao[a]#, Shengjie Zhang[a,b], Sumit Kumar[c,d], Heng Zhou[a], Qiong Xue[a,b], Wurunze Sun[a,b], Jian Zhou[a], Hua Xiang[a,b]#

[a]State Key Laboratory of Microbial Resources, Institute of Microbiology, Chinese Academy of Sciences, Beijing, China

[b]College of Life Sciences, University of Chinese Academy of Sciences, Beijing, China

[c]Enzyme and Microbial Biochemistry Lab, Department of Chemistry, Indian Institute of Technology, Delhi, India

[d]Amity Institute of Biotechnology, Amity University, Noida, Uttar Pradesh, India

Running Head: Comparative genomics of "*Candidatus* Nanohaloarchaeota"

#Address correspondence to: Hua Xiang, xiangh@im.ac.cn; Dahe Zhao, zhaodh@im.ac.cn

Dahe Zhao, Shengjie Zhang, and Sumit Kumar contributed equally to this work. Author order was determined in order of the time participating in the research.

Outputs for statistical analysis of mean comparison

1. data exhibition

```
> data <- read.csv("statistics_HGTector_ratio.csv", header = TRUE)
> data
            strain                    group Ratio_Halobacteria   ApI GC_content
1  AB_1215_Bin_137           AB_1215_Bin_137               0.00 8.104     32.355
2            NHA21 Nanohalalkaliarchaeaceae              47.86 5.035     56.090
3            NHA24     Nanoanaerosalinaceae              18.33 6.106     38.128
4            NHA20     Nanoanaerosalinaceae              15.38 5.690     41.204
5            NHA23     Nanoanaerosalinaceae              15.38 6.162     38.059
6            NHA-2     Nanoanaerosalinaceae              12.94 5.976     38.520
7          J07AB56           Nanosalinaceae              79.25 5.432     56.204
8            NHA25           Nanosalinaceae              57.80 4.865     48.481
9          MAG_AT22          Nanosalinaceae              49.22 5.186     44.314
10        SW_7_43_1          Nanosalinaceae              50.34 5.210     42.610
11          Nha-CHl          Nanosalinaceae              65.09 5.592     40.321
12     B1-Br10_U2g1          Nanosalinaceae              62.26 5.174     42.570
13           NHA26           Nanosalinaceae              46.25 5.255     40.691
14            M3_22          Nanosalinaceae              74.23 5.114     46.945
15          J07AB43          Nanosalinaceae              58.33 5.223     43.581
16           NHA29           Nanosalinaceae              50.47 4.917     44.824
17           LC1Nh           Nanosalinaceae              56.55 5.078     43.238
18             SG9           Nanosalinaceae              53.30 5.182     46.368
19    B1-Br10_U2g21          Nanosalinaceae              50.00 5.184     39.372
20           NHA-1           Nanosalinaceae              64.71 5.184     39.832
```

2. T-test for the ratio of HGTs

```
> # normally distributed test for the data of each factor level
> with(data, shapiro.test(Ratio_Halobacteria[group == "Nanosalinaceae"]))

        Shapiro-Wilk normality test

data:  Ratio_Halobacteria[group == "Nanosalinaceae"]
W = 0.917, p-value = 0.199

> with(data, shapiro.test(Ratio_Halobacteria[group == "Nanoanaerosalinaceae"]))

        Shapiro-Wilk normality test

data:  Ratio_Halobacteria[group == "Nanoanaerosalinaceae"]
W = 0.94045, p-value = 0.6571
```

```
> t.test(Ratio_Halobacteria ~ group, data = filter(data, group == c("Nanosalinaceae", "Nanoanaerosalinacea
e")), var.equal = TRUE)

        Two Sample t-test

data:  Ratio_Halobacteria by group
t = -5.1382, df = 7, p-value = 0.001341
alternative hypothesis: true difference in means between group Nanoanaerosalinaceae and group Nanosalinace
ae is not equal to 0
95 percent confidence interval:
 -63.74197 -23.56374
sample estimates:
mean in group Nanoanaerosalinaceae       mean in group Nanosalinaceae 
                          14.16000                           57.81286 

> t.test(filter(data, group == "Nanosalinaceae")$Ratio_Halobacteria, mu = 47.86, alternative = "two.side
d")

        One Sample t-test

data:  filter(data, group == "Nanosalinaceae")$Ratio_Halobacteria
t = 4.0355, df = 13, p-value = 0.001414
alternative hypothesis: true mean is not equal to 47.86
95 percent confidence interval:
 52.76419 64.06439
sample estimates:
mean of x 
 58.41429 

> t.test(filter(data, group == "Nanoanaerosalinaceae")$Ratio_Halobacteria, mu = 47.86, alternative = "two.
sided")

        One Sample t-test

data:  filter(data, group == "Nanoanaerosalinaceae")$Ratio_Halobacteria
t = -29.34, df = 3, p-value = 8.695e-05
alternative hypothesis: true mean is not equal to 47.86
95 percent confidence interval:
 11.99825 19.01675
sample estimates:
mean of x 
  15.5075 
```

In normality tests, p-values are more than 0.05, so the data of the two groups are normal distribution, and T-tests were performed.
In the T-tests, p-values are less than 0.05, so the differences are significant.

3. T-test for average isoelectric point

```
> # normally distributed test for the data of each factor level
> with(data, shapiro.test(ApI[group == "Nanosalinaceae"]))

        Shapiro-Wilk normality test

data:  ApI[group == "Nanosalinaceae"]
W = 0.90723, p-value = 0.1437

> with(data, shapiro.test(ApI[group == "Nanoanaerosalinaceae"]))

        Shapiro-Wilk normality test

data:  ApI[group == "Nanoanaerosalinaceae"]
W = 0.89973, p-value = 0.4297
```

```
> t.test(ApI ~ group, data = filter(data, group == c("Nanosalinaceae", "Nanoanaerosalinaceae")), var.equal
 = TRUE)

        Two Sample t-test

data:  ApI by group
t = 3.865, df = 7, p-value = 0.006171
alternative hypothesis: true difference in means between group Nanoanaerosalinaceae and group Nanosalinace
ae is not equal to 0
95 percent confidence interval:
 0.2152299 0.8936273
sample estimates:
mean in group Nanoanaerosalinaceae       mean in group Nanosalinaceae
                          5.833000                           5.278571

> t.test(filter(data, group == "Nanosalinaceae")$ApI, mu = 5.035, alternative = "two.sided")

        One Sample t-test

data:  filter(data, group == "Nanosalinaceae")$ApI
t = 3.1149, df = 13, p-value = 0.008208
alternative hypothesis: true mean is not equal to 5.035
95 percent confidence interval:
 5.081097 5.289760
sample estimates:
mean of x
 5.185429

> t.test(filter(data, group == "Nanoanaerosalinaceae")$ApI, mu = 5.035, alternative = "two.sided")

        One Sample t-test

data:  filter(data, group == "Nanoanaerosalinaceae")$ApI
t = 9.0073, df = 3, p-value = 0.002889
alternative hypothesis: true mean is not equal to 5.035
95 percent confidence interval:
 5.648376 6.318624
sample estimates:
mean of x
   5.9835
```

```
> t.test(filter(data, group == "Nanoanaerosalinaceae")$ApI, mu = 5.873, alternative = "two.sided") # for S
alinibacter ruber

	One Sample t-test

data:  filter(data, group == "Nanoanaerosalinaceae")$ApI
t = 1.0493, df = 3, p-value = 0.3711
alternative hypothesis: true mean is not equal to 5.873
95 percent confidence interval:
 5.648376 6.318624
sample estimates:
mean of x 
   5.9835 

> t.test(filter(data, group == "Nanoanaerosalinaceae")$ApI, mu = 6.223, alternative = "two.sided") # for S
piribacter salinus

	One Sample t-test

data:  filter(data, group == "Nanoanaerosalinaceae")$ApI
t = -2.2744, df = 3, p-value = 0.1075
alternative hypothesis: true mean is not equal to 6.223
95 percent confidence interval:
 5.648376 6.318624
sample estimates:
mean of x 
   5.9835 
```

In normality tests, p-values are more than 0.05, so the data of the two groups are normal distribution, and T-tests were performed.

In the T-tests between any two of the three groups, p-values are less than 0.05, so the differences are significant.

In the T-tests between "Nanoanaerosalinaceae" and two reference species, p-values are more than 0.05, so the differences are not significant.

4. Wilcoxon signed rank test for G + C content

```
> # normally distributed test for the data of each factor level
> with(data, shapiro.test(GC_content[group == "Nanosalinaceae"]))

        Shapiro-Wilk normality test

data:  GC_content[group == "Nanosalinaceae"]
W = 0.86831, p-value = 0.03973

> with(data, shapiro.test(GC_content[group == "Nanoanaerosalinaceae"]))

        Shapiro-Wilk normality test

data:  GC_content[group == "Nanoanaerosalinaceae"]
W = 0.73649, p-value = 0.02863

> wilcox.test(GC_content ~ group, data = filter(data, group == c("Nanosalinaceae", "Nanoanaerosalinacea
e")), var.equal = TRUE)

        Wilcoxon rank sum exact test

data:  GC_content by group
W = 3, p-value = 0.3333
alternative hypothesis: true location shift is not equal to 0

> wilcox.test(filter(data, group == "Nanosalinaceae")$GC_content, mu = 56.090, alternative = "two.sided")

        Wilcoxon signed rank exact test

data:  filter(data, group == "Nanosalinaceae")$GC_content
V = 1, p-value = 0.0002441
alternative hypothesis: true location is not equal to 56.09

> wilcox.test(filter(data, group == "Nanoanaerosalinaceae")$GC_content, mu = 56.090, alternative = "two.si
ded")

        Wilcoxon signed rank exact test

data:  filter(data, group == "Nanoanaerosalinaceae")$GC_content
V = 0, p-value = 0.125
alternative hypothesis: true location is not equal to 56.09
```

In normality tests, p-values are less than 0.05, so the data of the two groups are not a normal distribution, and Wilcoxon tests were performed.
In the Wilcoxon test between "Nanosalinaceae" and NHA21, a p-value is less than 0.05, so the difference between them is significant. For the other two tests, the differences are not significant.
